# From Controlled Scenarios to the Real World: Cross-Domain Degradation Pattern Matching for All-in-One Image Restoration

**DOI:** 10.34133/research.1191

**Published:** 2026-03-27

**Authors:** Junyu Fan, Chuanlin Liao, Endi Xie, Dongyue Guo, Xiaolin Gou, Duan Wei, Junyang Hu, Yi Lin

**Affiliations:** ^1^ College of Computer Science, Sichuan University, Chengdu, China.; ^2^ National Key Laboratory of Fundamental Science on Synthetic Vision, College of Computer Science, Sichuan University, Chengdu, China.

## Abstract

As a fundamental imaging task, All-in-One Image Restoration (AiOIR) aims to achieve image restoration caused by multiple degradation patterns via a single model with unified parameters. However, existing methods typically rely on sample-wise supervision, which tends to entangle degradation features with image content. Furthermore, the inevitable distribution shift between training data (source domain) and real-world samples (target domain) weakens degradation awareness, severely limiting the generalization capability of models in real-world scenarios. To address this, a Unified Domain-Adaptive Image Restoration (UDAIR) computer vision model is proposed by achieving the transition from learning unstable local features to learning robust universal prototypes. To decouple degradation from content, a Cross-Sample Contrastive Learning mechanism is implemented by a codebook-based module. By contrasting samples with shared degradations but diverse content, the proposed model learns discrete embeddings as degradation prototypes. Furthermore, to actively bridge the distribution gap during inference, a correlation alignment-based test-time adaptation mechanism is designed to dynamically pull drifting target features toward their corresponding degradation cluster centers to effectively eliminate residual alignment discrepancies. Experimental results on 10 open-source datasets demonstrate that UDAIR achieves new state-of-the-art performance for the AiOIR task, in which each technical module contributes to the desired performance improvement. Most importantly, the feature cluster validates the degradation identification under multiple degradation patterns, and qualitative comparisons showcase robust generalization to real-world scenarios.

## Introduction

**Fig. 1.**
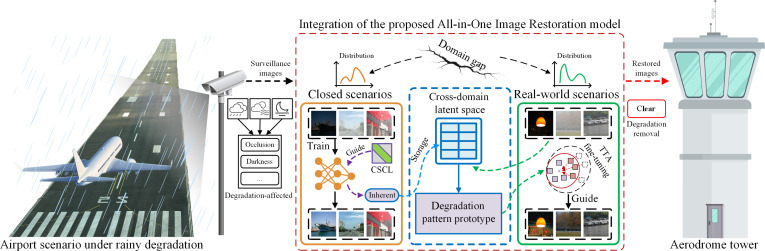
Application of the proposed model to restore clear images from degraded environments (e.g., rainy, hazy, and low-light conditions) in the airport surveillance system. To bridge the gap between closed scenarios and real-world scenarios, the proposed model establishes a cross-domain latent space via a codebook, which stores the shared intrinsic features of degradation patterns optimized through cross-sample contrastive learning (CSCL). These features serve as prototypes of degradation patterns, effectively leveraging the knowledge and priors learned from closed scenarios. Test-time adaptation (TTA) is further employed to fine-tune the model for each sample, compensating for errors introduced during cross-domain matching. This strategy mitigates performance decline caused by domain shifts in degradation pattern recognition and guides the network to restore high-quality images from low-quality inputs affected by various degradations.

All things resolved within a single framework is the ultimate goal for data-driven tasks, as well as the image restoration (IR) task in this work. As the foundation of visual intelligence, the IR task serves as a critical technology to restore degraded visual data into clear and detailed representations, which further provides essential perceptual support for computer vision systems. As the core capability of artificial intelligence to perceive the physical world, clear and detailed visual images in a computer vision system directly determine the potential for humans to break through perceptual boundaries and expand decision-making dimensions. In past studies, IR approaches predominantly focused on individual degradation patterns in closed and controlled scenarios, and required different models or parameter weights for different cases (denoising [1], dehazing [2], low-light [3], etc.). However, images captured in real world are with diverse and complex degradation patterns, which presents a data gap from the ideal case in controlled scenarios [4]. In this context, to achieve the IR task with multiple degradations, a set of models is required to be separately built and trained for each degradation, which reduces the generalizability and robustness in practical applications. For instance, in the airport surface surveillance scenarios under hazing, raining, and low-light conditions, different IR models are deployed and sequentially performed to obtain a clear and detailed monitoring image to support the downstream tasks, which not only burdens the total resource requirements and system latency but also causes unexpected cascaded failures (each IR model has their own unique task objective). Therefore, an All-in-One Image Restoration (AiOIR) framework has emerged as a promising solution to tackle images with multiple degradation patterns using only a single model with unified parameters [5]. The AiOIR is expected to provide a general solution for degraded IR in complex environments, such as autonomous driving [6], unmanned aerial vehicle [7], underwater robotics [8], and other applications [9–11]. Figure 1 illustrates the application of the proposed AiOIR model to automatically identify degradation patterns and restore clear images from different degraded environments in airport surveillance systems.

Currently, IR methods can be divided into single degradation image restoration (SDIR), multiple degradation image restoration (MDIR), and AiOIR, as shown in Fig. S1. The SDIR methods, such as shown in Refs. [12–14], are typically designed for specific tasks and are particularly effective at handling known degradation patterns. However, these methods suffered from poor performance facing unseen degradation distributions of degradation patterns. The MDIR methods, such as presented in Refs. [15–18], attempt to develop a unified framework to flexibly cope with various degradation with degradation-related model weights. Although MDIR methods achieve higher generalizability for IR tasks, they still require the degradation patterns as prior to perform preselected IR operations in practical applications. Recently, AiOIR methods, such as indicated in Refs. [19–23], are proposed to simultaneously address multiple diverse degradation tasks using a single framework with unified parameter weights, thereby providing both generality and practicality in real-world tasks.

In general, the key to AiOIR approaches depends on the ability to effectively identify degradation patterns that are usually not predefined and cannot be determined in advance. In addition, in real-world scenarios, data gap of different domains inevitably deteriorates the performance of existing methods with degradation prior assumptions from close scenarios datasets [5], indicating the dual challenges of bias in identifying degradation patterns and domain distribution shift [24], as shown below:

• In recent works, prompt-based learning [21], contrastive learning [25], and multimodal [26] were proposed and improved to enhance the representation capabilities of complex degradations. However, due to single-sample optimization, these methods tend to capture sample-wise features with limited ability to represent the actual degradation pattern. Therefore, instead of capturing the generalizable physical properties of the degradation, they suffer from overfitting problems on sample-specific semantic information, which fails to model shared degradation characteristics across samples, resulting in compromised identification capabilities, and further deteriorate the final restoration performance.
• Existing methods typically assume identical feature distributions across training and testing samples. However, statistical discrepancies between the source and target domains inevitably render the learned degradation pattern identifiers ineffective on unseen data. Facing this distribution shift, it is hard to directly transfer the learned representations from the source domain data to the target domain data, in which any failures of degradation identification can confuse the restoration model with incorrect tasks and further impact the final performance. Actually, existing methods can only obtain desired performance in training closed scenarios (source domain), while suffering from poor generalization ability in environments with different feature distributions (target domain). In addition, due to the diversity of real-world scenarios, training samples and real-world samples are usually unseen from each other, which substantially increases the difficulty of cross-domain adaptation.

To address the aforementioned issues, a Unified Domain-Adaptive Image Restoration (UDAIR) framework is proposed to effectively utilize the knowledge and priors learned from the source domain during inference with real-world scenario data. The core idea of the proposed UDAIR redefines the AiOIR task, i.e., solving complex interdomain differences by building and matching cross-domain degradation pattern prototypes. To enhance the degradation perception identification across different data domains, a Cross-Sample Contrastive Learning (CSCL) mechanism is proposed to guide the model to explore the shared intrinsic representations of a certain degradation pattern across diverse images. CSCL groups the degradation features of multiple samples by task and generates positive sample pairs with the same degradation pattern while exhibiting different degradation manifestations. To this end, a codebook-based Degradation Aware and Analysis Module (DAAM) is designed to establish a cross-domain latent space to represent degradation pattern prototypes by learning shared intrinsic priors of degradation patterns rather than sample-wise local features unrelated to degradation, which is expected to be applicable in identifying this degradation in unseen data domains. For a certain sample, the prototype of the codebook is selected to match the prototype that most closely corresponds to the degradation pattern, leveraging the intrinsic properties of degradation prototypes to build a bridge across domains and alleviate the data distribution shift.

Considering the inherent distribution discrepancies of images between source and target domains, in this work, a correlation alignment test-time adaptation (TTA) strategy is proposed to perform the online readjustment via progressive alignment based on the sample-wise features, which helps mitigate matching errors caused by interdomain distribution discrepancies. Existing TTA approaches [27,28] were typically optimized for decision boundary certainty, which fails to capture the fine-grained distributional statistics required for pixel-dense IR. Moreover, many adaptation strategies rely on the simultaneous availability of source and target samples, limiting their practical deployment. Considering the unique challenges of IR, the proposed TTA strategy is designed to align feature distributions between different data domains. By regarding the degradation feature cluster centers learned in the source domain as anchors, correlation alignment is employed to rectify the second-order statistics of target domain features, with the objective of establishing a shared degradation representation. This approach is intended to bridge cross-domain discrepancies by progressively pulling target features toward their corresponding anchors, providing more discriminative degradation features to benefit the restoration in complex real-world scenarios.

To validate the proposed UDAIR model, a total of 5 common IR tasks, concerning denoising, dehazing, deraining, low-light image enhancement (LLIE), and underwater image enhancement (UIE), are selected to conduct comparison experiments. For each task, 2 different datasets serve as the source and target domains, respectively. Recent competitive baselines are also applied to evaluate the model performance in terms of both common IR metrics and the specific task-related measurements. Extensive experimental results demonstrate that the UDAIR outperforms other baselines and achieves new state-of-the-art performance for the AiOIR task. In objective evaluations, the proposed method outperforms recent state-of-the-art approaches by an average of 4.3%, with a notable improvement of around 5% on the target domain. Most importantly, the visualizations of feature cluster validate the degradation identification under multiple degradation patterns, and qualitative comparisons showcase robust generalization to real-world scenarios, which finally supports the innovative motivations in this work.

In summary, this work contributes the AiOIR in the following ways:

• A UDAIR framework is proposed to effectively mitigate performance reduction caused by distribution discrepancies, which leverages learned knowledge and priors from the source domain to construct a cross-domain latent space to match degradation patterns in the target domain. The UDAIR model enhances both the degradation pattern identification and restoration performance in real-world scenarios.
• A CSCL mechanism is proposed to learn informative representations for the intrinsic nature of image degradation by constructing sample pairs from different samples with certain degradation patterns, which is also able to suppress the interference features to further enhance the identification capacity of the degradation patterns.
• A codebook-based domain adaptation strategy is proposed to establish a shared cross-domain feature space by preserving learned prototypes of degradation patterns, based on which the TTA is combined to perform the dynamic fine-tuning for the UDAIR model. The domain adaptation aims to address distribution shifts of the cross-domain samples, and the decreasing Kullback–Leibler (KL) divergence further demonstrates the effectiveness of the proposed strategy.
• Extensive experimental results demonstrate that the proposed model achieves new state-of-the-art performance in an AiOIR way for all 5 tasks on both 10 open-source closed and real-world scenario datasets, in terms of all the proposed metrics. The in-depth analysis also supports the research motivation in this work and validates the potential for real-world applications.

## Results

### Task overview

Given a degraded image $y\in {\mathbb{R}}^{H\times W\times C}$ and the corresponding clean image $x\in {\mathbb{R}}^{H\times W\times C}$, the degradation process can be modeled as:

y=Dxd, (1)

where D is the degradation function and d is the degradation pattern.

For SDIR and MDIR methods, separate models are required to be trained for specific degradation patterns or with a preselected degradation pattern to restore a clean image $\hat{x}$, as in:

$${\hat{x}}_{i}=M_{\theta_{i}}^{k}\left(y,\;d_{i}\right),$$ (2)

where $M^{k}$ is the trainable model with known degradation patterns; $\theta_{i}$ is optimized only for a certain degradation pattern $d_{i}$. Therefore, m sets of model weights $\left\{\theta_{1},\;\theta_{2},\ldots ,\;\theta_{m}\right\}$ are optimized with different training samples for each degenerate pattern $\left\{d_{1},\;d_{2},\ldots ,\;d_{m}\right\}$.

In this work, AiOIR recovers the clean image with degradation awareness and adaptive restoration by a single model weight, in which the model can automatically detect the degradation patterns from y by implicit degradation perception:

$$\hat{x}=M_{\theta }^{s}\left(y\right)=R\left(y,\;I\left(y,\;\theta_{a}\right),\;\theta_{r}\right),$$ (3)

where $M^{s}$ is the trainable model on the source domain; R is the restoration operation; I is the degradation identification operation; $\theta =\left\{\theta_{a},\;\theta_{r}\right\}$ is unique and independent of degradation pattern d; $\theta_{a}$ denotes the weights of degradation identification; and $\theta_{r}$ denotes the weights of IR.

In addition, the generalization challenge of the AiOIR task in real-world scenarios is formulated as a domain adaptation problem. During the model inference for target domain samples, the proposed UDAIR framework introduces TTA to optimize the Domain Adaptation Module (DAM), as in:

$$\theta_{da}=T\left(\theta_{da}^{\prime },\;A_{c}\right)=\theta_{da}^{\prime }-\eta \cdot \nabla_{\theta_{da}^{\prime }}L_{TTA}\left(\theta_{da}^{\prime },\;A_{c}\right),$$ (4)

where T is the TTA mechanism; $\theta_{da}$ is the weights of the DAM, which are activated and updated only during the testing procedure on target domain samples; $L_{TTA}$ denotes the loss computed during the TTA procedure; $A_{c}$ denotes the anchor of the degradation pattern, which is the cluster center of the degradation features in the source domain; η denotes the learning rate; and ∇ denotes the gradient operator.

Finally, the test procedure in the target domain can be described as:

$${\hat{x}}^{\prime }=M_{\theta }^{t}\left(y\right)=R^{\prime }\left(y,\;I\left(y,\;\theta_{a},\;T\left(\theta_{da},\;A_{c}\right)\right);\;\theta_{r}\right),$$ (5)

where $M^{t}$ and $R^{\prime }$ are the trainable model and restoration operation on the target domain, respectively.

### Dataset and data preprocessing

In this work, 4 common real-world imaging challenges (sensor noise, aerosol scattering, rain streak occlusion, and low-light) are focused to validate the proposed model, in which each corresponds to a distinct physical degradation process. In general, the mentioned challenges are inherently caused by the interplay of imaging hardware limitations (e.g., noise) and complex environmental conditions (e.g., haze, rain, and low-light), which concern the denoising, dehazing, deraining, and low-light image enhancement tasks for critical computer vision systems. In addition, the underwater image enhancement task is also considered to explore the ability of the model to handle complex degradations. In the underwater environment, most atmospheric degradations result from particle scattering and absorption, in which noticeable color attenuation, nonuniform lighting, and low contrast are also presented due to the water turbidity. The UIE task requires the IR model not only to remove occlusions but also to perform accurate spectral and color corrections. It is believed that the mentioned task design can provide a more demanding challenge to formulate a solid evaluation across diverse degradations.

For each mentioned IR task, 2 independent datasets are selected to represent the data distribution in the source and target domains, respectively, which further explores the capacity of the model to address the distribution shifts among different domains (datasets). In this context, a total of 10 open-source datasets are determined to conduct the comparison experiments, as shown below:

Source domain datasets: Smartphone Image Denoising Dataset (SIDD) [29] for denoising, Outdoor Training Set (OTS) [30] for dehazing, RealRain-1k [31] for deraining, Low-Light dataset (LOL) [32] for LLIE, and Underwater Image Enhancement Benchmark (UIEB) [33] for UIE.

Target domain datasets: Polyu [34] for denoising, Unannotated Real-world Hazy Images (URHI) [30] for dehazing, Large-scale High-quality Paired real rain benchmark (LHP-Rain) [35] for deraining, Low-light Image Enhancement (LIME) [36] for LLIE, and UFO-120 [37] for UIE.

In general, the source domain datasets are selected to approximate real-world conditions by either synthetic or real collected images, while the samples in target domain datasets are collected from real scenes to imitate the distribution shifts, which is expected to formulate a robust controllable domain mapping and alignment.

Since the proposed UDAIR model is trained to perform the AiOIR on all 5 IR tasks, it is required to organize a balanced dataset across different degradation patterns to enhance the model training and convergence. Considering different sample numbers in the source domain dataset, the full dataset for RealRain-1k (deraining), LOL (LLIE), and UIEB (UIE) are applied to train the proposed model, including 2,016 deraining (1,568 for training and 448 for testing), 1,500 LLIE (1,300 for training and 200 for testing), and 890 UIE (700 for training and 190 for testing) images, respectively. For the SIDD denoising dataset, a total of 160 images for 10 scenes were used. Due to extremely high-resolution images (i.e., over 4,000×4,000), all raw images are cropped into the common 512×512, which greatly increases the size of the dataset. To organize a balanced dataset across different degradation patterns, for each scene, 4 raw images are selected to formulate the dataset, in which 8 scenes are for training and the remaining 2 scenes are for testing. Finally, a total of 2,148 images are obtained for the denoising dataset (1,812 for training and 336 for testing). As to the OTS dehazing dataset, for all the 2,061 scenes, one image for each scene is selected to finally obtain 2,061 dehazing samples (1,500 for training and 561 for testing). For the target domain datasets, the full version of all the datasets is applied to the TTA and test. All the source domain datasets are applied to training with data augmentation (random flipping, random rotation, and random cropping) to enhance generalization, and all the target domain datasets are applied to testing without any data augmentation. During the training procedure, images are randomly cropped to a resolution of 128, and during testing, images are resized to 512 (source domain datasets) and 256 (target domain datasets) resolutions to avoid deployment issues caused by hardware limitations.

### Evaluation metrics

For full-reference datasets (i.e., SIDD, OTS, RealRain-1k, LOL, UIEB, Polyu, and LHP-Rain), the common full-reference metrics Peak Signal-to-Noise Ratio (PSNR) and Structure Similarity Index Measure (SSIM) [38] are selected to evaluate the IR performance by measuring the discrepancy and similarity between the restored images and their clean reference. For nonreference datasets (including URHI, LIME, and UFO-120), the task-related evaluation metrics are selected to evaluate the IR performance, as summarized as

• For the dehazing task, the Fog Aware Density Estimator (FADE) is widely used to estimate haze density in an image by leveraging statistical features related to fog. The FADE has been frequently adopted in dehazing studies [14], to provide a nonreference assessment of haze severity.
• For the LLIE task, the Natural Image Quality Evaluator (NIQE) is a popular nonreference metric to quantify image quality based on deviations from statistical regularities observed in natural scenes. The NIQE has been extensively employed in LLIE works [12]. In addition, the Blind/Referenceless Image Spatial Quality Evaluator (BRISQUE) is also frequently applied to evaluate perceptual distortions perceptible to the human visual system.
• For the UIE task, the Underwater Image Quality Measurement (UIQM) and Underwater Colour Image Quality Evaluation (UCIQE) are standard evaluation metrics commonly adopted to assess the overall visual quality of enhanced underwater images [39].

### Baselines

In this work, the following recent AiOIR models are selected as the comparative baselines to comprehensively validate the proposed model. For all the baselines, default hyperparameter settings are the same as the corresponding references to implement the IR tasks.

• AirNet [25] employs contrastive learning to extract degradation representations from degraded images to guide the network in restoring clean images.
• ROP^+^ [40] assumes that degradation can be modeled as a rank-one matrix overlaid on a clean background. ROP^+^ decomposes the degraded image into a low-rank background (clean image) and a sparse degradation layer (e.g., haze) to achieve IR.
• PromptIR [21] generates task-specific prompt vectors through a lightweight encoder, dynamically guiding the model to adapt to various restoration tasks.
• CAPTNet [20] directly integrates learnable prompts into the network architecture and separately incorporates local and nonlocal pixel interactions to enable adaptive IR.
• DiffUIR [41] adopts a diffusion model framework, integrating the progressive denoising process of the diffusion model with the feature selection capabilities of a U-Net-like architecture to iteratively generate clear images.
• DFPIR [42] employs degradation-aware feature perturbations to adjust the feature space, to better align with a unified parameter space and thereby to mitigate task interference.
• AdaIR [19] analyzes the frequency features to separate the frequency subbands affected by different degradations (e.g., high-frequency noise and low-frequency haze), enabling targeted modulation of features for restoration.

### Experimental configuration

In this work, to address the issue of imbalanced training samples across tasks, the samples of each task are randomly resampled to match the size of the largest dataset. In each iteration, an equal number of samples from all 5 tasks are randomly selected to form a mini-batch, which is then applied to perform joint training. The DAAM projects each sample into a 96-dimensional space to better perceive and learn degradation patterns, which subsequently guide the backbone for IR. The backbone is configured with a base dimensionality of 24 and progressively processes features by coupling spatial downsampling and upsampling with corresponding changes in feature dimensionality. At the bottleneck layer, the backbone reaches 8 times of the base dimensionality with an 8× downsampling rate. Each sample produces 24,576 features under the CSCL constraint. The TTA and DAM are employed during testing time on target domain samples; the step of TTA is set to 5, and the DAM adopts the same dimensionality configuration as the decoder to ensure feature compatibility. The proposed method is trained with an AdamW optimizer at an initial learning rate of 1 × 10^−4^, and a cosine annealing schedule is employed to gradually reduce the learning rate throughout the training process.

All experiments are conducted in the same experimental environment to ensure fairness. The hardware includes an Intel Xeon Gold 5318Y CPU @ 2.10 GHz, 4*NVIDIA GeForce RTX 4090 GPUs, and 512 GB of memory. Experiments are performed on Ubuntu 20.04 using the open-source PyTorch 2.4.0 framework.

### Results and quantitative analysis

#### Performance on the source domain

Table 1A presents the performance comparison of the different methods on the source domain datasets in terms of objective metrics, which provides the effectiveness of the comparative models before domain adaptation (mainly concerns the degradation pattern identification in this work). In general, the proposed method harvests the best SSIM and PSNR on denoising, dehazing, deraining, and LLIE, with a substantial improvement on the deraining task. On the UIE task, the proposed model also achieves a comparable performance with only a slight performance gap. The mentioned results demonstrate that the proposed model provides desired learning and inference capabilities on the source domain. Fortunately, except for the obtained numerical performance improvements, the proposed model has the ability to consistently cope with all degradation patterns, validating its universal degradation pattern identification and robust image reconstruction. In addition, compared with the baselines, the proposed model exhibits an excellent balance between performance and computational complexity. As shown in Fig. S4, the proposed model not only achieves the best performance but also maintains a satisfactory level of computational efficiency. To be specific, the following conclusion can be obtained based on the reported experimental results:

1. The proposed model obtains enhanced PSNR and SSIM metrics. The higher PSNR indicates that the proposed model can recover the degraded images with minimized pixel-level errors by removing the degradations and suppressing local distortions, such as noise, artifacts, and color blotches. Meanwhile, the better SSIM confirms that the proposed model can also provide expected perceptual similarity by preserving texture and structural information. Based on the combination of the 2 metrics, the proposed model can provide higher restoration quality over diverse degradations. The results can be attributed to the fact that, compared to the local degradation features from single samples (such as AirNet and PromptIR), the proposed CSCL strategy captures more representative and shared degradation patterns in the feature space to support degradation identification and recovery, even in structurally complex scenes.
2. In the denoising task, the image noise is typically represented as high-frequency features, i.e., randomly distributed pixel perturbations, disrupting smooth regions and obscuring fine edge textures. The proposed model obtains superior objective scores by effectively suppressing random noise and restoring texture details and structural integrity. In contrast, in the dehazing task, haze degradation manifests as low-frequency global veiling caused by scattered light, which lowers image contrast and fades distant details. The proposed model still achieves the highest PSNR and SSIM with the closest similarity to the reference image. Meanwhile, the deraining task requires the restoration model to suppress sparse and high-frequency rain streaks and recover the occluded background. Although DFPIR achieves the best deraining performance among the comparison methods, the proposed method still obtains approximately 2.5 dB PSNR improvement. It is noted that the proposed model harvests 35.193 dB PSNR, with an about 5-dB absolute improvement over the best overall baseline (i.e., AdaIR). The results on the 3 tasks confirm the ability to handle both the low- and high-frequency features, as well as sparse degradation distribution, i.e., removing noise/rain/haze and restoring fine details and color consistency by the effective identification of degradation patterns.
3. It can be seen from the results that, in the LLIE and UIE tasks, all comparative methods suffer from PSNR and SSIM reduction due to inherent task natures, including severe degradations, limited dynamic range, and complex interplay of scattering and color cast, which also support the motivation to validate the proposed model under complex real-world conditions. Fortunately, the proposed model also obtains the best performance over other baselines on the LLIE task, i.e., balanced brightness enhancement with preservation for coherence of edges in dark regions and highlight details. As to the UIE task, the proposed model achieves comparable performance with a slight gap due to coupled degradations (noise, scattering, uneven illumination, etc.). It is believed that the performance gap results from the highly complex degradation of underwater environments, i.e., the noise, scattering, and uneven illumination correspond to the other tasks. The proposed model may focus on learning separate degradation representations and clustering them accordingly due to the sample size for the other tasks far exceeding that of the UIE task. As a result, complex and entangled degradation patterns may be decoupled, and the model tends to capture only the dominant underwater features, such as strong blue–green color casts for underwater environments (as illustrated in the visual comparison in the “Visualization and qualitative analysis” section).

**Table 1.** Objective performance comparison of different methods in comparative analysis (A and B) and different variants in ablation study (C and D), where the best (second-best) performance is indicated in boldface (italics); ↑ indicates that higher values are better, and ↓ indicates that lower values are better

(A) Quantitative comparison of different methods on the source domain											
Method	Venue	Denoising		Dehazing		Deraining		LLIE		UIE	
		SSIM↑	PSNR↑	SSIM↑	PSNR↑	SSIM↑	PSNR↑	SSIM↑	PSNR↑	SSIM↑	PSNR↑
AirNet	CVPR’22	0.938	38.505	0.911	25.716	0.843	26.691	0.748	15.443	0.804	18.938
ROP^+^	TPAMI’23	0.116	7.226	0.634	13.761	0.376	13.914	0.582	14.855	0.716	16.012
PromptIR	NeurIPS’23	0.942	38.871	0.926	27.398	0.821	27.502	0.834	19.922	0.834	21.623
CAPTNet	TCSVT’24	0.913	37.270	0.932	26.301	0.686	23.327	0.709	19.226	0.821	20.423
DiffUIR	CVPR’24	*0.950*	*39.777*	0.930	27.703	0.872	30.053	*0.867*	21.279	**0.840**	**21.962**
DFPIR	CVPR’25	0.908	37.373	0.926	28.142	*0.921*	*32.852*	0.865	21.491	0.828	21.547
AdaIR	ICLR’25	0.948	39.415	*0.934*	*28.737*	0.875	30.251	0.832	*21.509*	*0.836*	*21.681*
UDAIR	Ours	**0.952**	**40.101**	**0.938**	**29.715**	**0.945**	**35.193**	**0.872**	**22.671**	0.833	21.208
(B) Quantitative comparison of different methods on the target domain											
Method	Venue	Denoising		Dehazing		Deraining		LLIE		UIE	
		SSIM↑	PSNR↑	FADE↓		SSIM↑	PSNR↑	NIQE↓	BRISQUE↓	UIQM↑	UCIQE↑
AirNet	CVPR’22	0.744	18.426	1.640		0.773	19.054	5.316	16.383	2.603	0.612
ROP^+^	TPAMI’23	0.477	12.913	**0.593**		0.556	14.718	5.290	18.196	**3.051**	**0.627**
PromptIR	NeurIPS’23	*0.873*	*26.581*	1.658		0.825	21.736	**4.714**	15.831	2.749	0.618
CAPTNet	TCSVT’24	0.783	22.211	1.664		0.798	20.670	5.388	21.877	2.746	0.610
DiffUIR	CVPR’24	0.777	21.254	1.722		0.765	20.490	6.127	*15.054*	*2.871*	0.618
DFPIR	CVPR’25	0.868	25.224	1.744		*0.837*	*22.284*	4.951	16.096	2.864	0.616
AdaIR	ICLR’25	*0.873*	26.156	1.629		0.829	21.183	4.850	15.751	2.717	0.617
UDAIR	Ours	**0.883**	**27.204**	*1.585*		**0.854**	**23.765**	*4.814*	**13.450**	2.811	*0.626*
(C) Ablation study on the source domain											
Variant	Venue	Denoising		Dehazing		Deraining		LLIE		UIE	
		SSIM↑	PSNR↑	SSIM↑	PSNR↑	SSIM↑	PSNR↑	SSIM↑	PSNR↑	SSIM↑	PSNR↑
Baseline	Ablation	*0.933*	*38.324*	0.928	27.602	*0.893*	*30.708*	0.800	19.397	0.816	20.332
Without CSCL		0.929	38.021	*0.929*	*27.669*	0.870	30.161	*0.824*	*20.045*	**0.834**	**21.266**
Without codebook		0.929	37.782	0.928	27.640	0.866	30.243	0.771	18.464	0.829	20.559
Full model		**0.952**	**40.101**	**0.938**	**29.715**	**0.945**	**35.193**	**0.872**	**22.671**	*0.833*	*21.208*
(D) Ablation study on the target domain											
Variant	Venue	Denoising		Dehazing		Deraining		LLIE		UIE	
		SSIM↑	PSNR↑	FADE↓		SSIM↑	PSNR↑	NIQE↓	BRISQUE↓	UIQM↑	UCIQE↑
Baseline	Ablation	0.841	24.992	1.871		0.809	20.919	5.278	16.811	2.496	0.611
Without CSCL		*0.872*	*26.836*	*1.609*		*0.844*	*22.784*	5.062	14.929	2.692	0.618
Without codebook		0.866	26.549	1.716		0.835	21.341	5.175	15.449	2.549	0.615
Without TTA		0.871	26.768	1.638		0.843	22.674	*4.899*	*14.750*	*2.694*	*0.621*
Without domain adaptation		0.844	25.718	1.761		0.826	21.182	5.143	15.958	2.545	0.610
Full model		**0.883**	**27.204**	**1.585**		**0.854**	**23.765**	**4.814**	**13.450**	**2.811**	**0.626**

CSCL, Cross-Sample Contrastive Learning; LLIE, low-light image enhancement; TTA, test-time adaptation; UIE, underwater image enhancement; UDAIR, Unified Domain-Adaptive Image Restoration

In summary, the results on the source datasets confirm the performance in terms of both the pixel-level errors and perceptual similarity, which validates the proposed model to cope with diverse degradations (low and high frequency, and sparse features) and supports the motivation of performance evaluation across different tasks.

#### Performance on the target domain

Table 1B reports the quantitative results of comparative methods on the target domain datasets. As the target domain serves as a strictly unseen cross-domain test set, these results are pivotal for evaluating the generalization capability of each model (for the domain adaptation in this work). Across the 5 designed tasks, the proposed model outperforms the baselines in most cases, validating the effectiveness of its domain adaptation strategy in cross-domain scenarios. Compared to baselines without explicit adaptation mechanisms, the proposed model consistently achieves substantial performance improvements on the target domain, indicating that the adaptation scheme has the ability to contribute the desired cross-domain transferability.

In general, the source domain datasets provide a relatively controlled experimental environment, where degradation patterns, capture conditions, and scene content remain consistent within the training sets. As a result, most models can effectively leverage learned statistical features to achieve expected PSNR and SSIM improvements. As the results of target datasets, all comparative methods suffer from considerable performance reduction by the mentioned distribution shifts. Fortunately, the proposed model provides desired performance superiority with strong cross-domain robustness. The results also support the core motivation in this work, i.e., learning universal and intrinsic degradation patterns during training and performing domain adaptation mechanisms at test time to achieve the generalizability from controlled scenarios to the real world.

1. Based on the selected datasets, both the denoising and deraining datasets provide degraded images along with corresponding clean references, allowing quantitative evaluation by the PSNR and SSIM. The reported results demonstrate that the proposed model achieves the highest PSNR and SSIM values, indicating the smallest pixel-level and structural errors compared to the reference images without degradation. Compared to the objective scores in source domain datasets, the best baseline AdaIR has about 13 dB in PSNR and 0.1 in SSIM absolute reduction, which indicates higher pixel-level errors and limited ability to reconstruct local structures in the target domain. Under the same challenging conditions, the proposed model harvests the best performance with 27.204 dB/0.883 for denoising and 23.765 dB/0.854 for deraining, with lower performance reduction (7, 12 dB in PSNR and 0.06, 0.09 in SSIM, respectively). This further suggests that the proposed model can not only remove more random noise and rain artifacts at the pixel level but also preserve scene structure and detail continuity more effectively.
2. Due to the lack of paired reference images, the performance of the dehazing, low-light, and underwater tasks is measured by nonreference metrics to represent the effectiveness of removing degeneration and the intraimage coherence and harmonies. In general, the proposed model harvests the best performance among the learning-based baselines (except ROP^+^). To be specific, compared to the most competitive baseline, AdaIR, the proposed model achieves 2.7% FADE improvements (from 1.629 to 1.585), 0.7% and 17% NIQE and BRISQUE for the LLIE task, and 3.4% and 1.4% UIQM and UCIQE for the UIE task, respectively. The results also validate the proposed model in achieving the IR with desired image naturalness, sharpness, contrast, noise suppression, and color accuracy and saturation. Most importantly, compared to methods without explicit adaptation mechanisms, the proposed model consistently achieves substantial performance improvements on the target domain, indicating that the adaptation scheme has the ability to contribute desired cross-domain transferability.
3. It can also be seen from the results that the model-based ROP^+^ baseline presents a different trend between the source and target domain datasets, as well as the reference and nonreference evaluation metrics. Specifically, the ROP^+^ suffers from the worst reference metrics in the source dataset, with a huge gap with other learning-based methods (0.116 vs. 0.952 SSIM and 7.226 vs. 40.101 PSNR for the proposed model). Since PSNR directly quantifies pixel-level fidelity, such low values indicate that the ROP^+^ exhibit substantial deviations from the reference images, indicating ineffective restoration of structural details. Conversely, the ROP^+^ obtains the best nonreference metrics for the dehazing and UIE task on the target domain dataset, also presenting a huge gap: 0.593 vs. 1.585 FADE, and 3.051 vs. 2.811 UIQM. However, it is noted that, without reference image validations, nonreference metrics are often biased toward specific statistical features and only partially reflect restoration quality. As can be seen from the visual comparisons, the high scores still fail to obtain better visual perception. Attributed to the learning-free paradigm using sparse-low-rank decomposition, the ROP^+^ is only applicable for specific tasks (dehazing and underwater in this work), which fails to handle complex degradations, such as texture noise, randomness, or lighting variation and finally leads to limited applicability for certain tasks, as well as poor objective metrics.

In summary, the experimental results on both the source and target domains in terms of the reference and nonreference metrics validate the proposed model in coping with the diverse degradation patterns across extensive experimental conditions, which further supports the research motivation of this work, i.e., enhanced ability of degradation identification and domain adaptation.

### Visualization and qualitative analysis

**Fig. 2.**
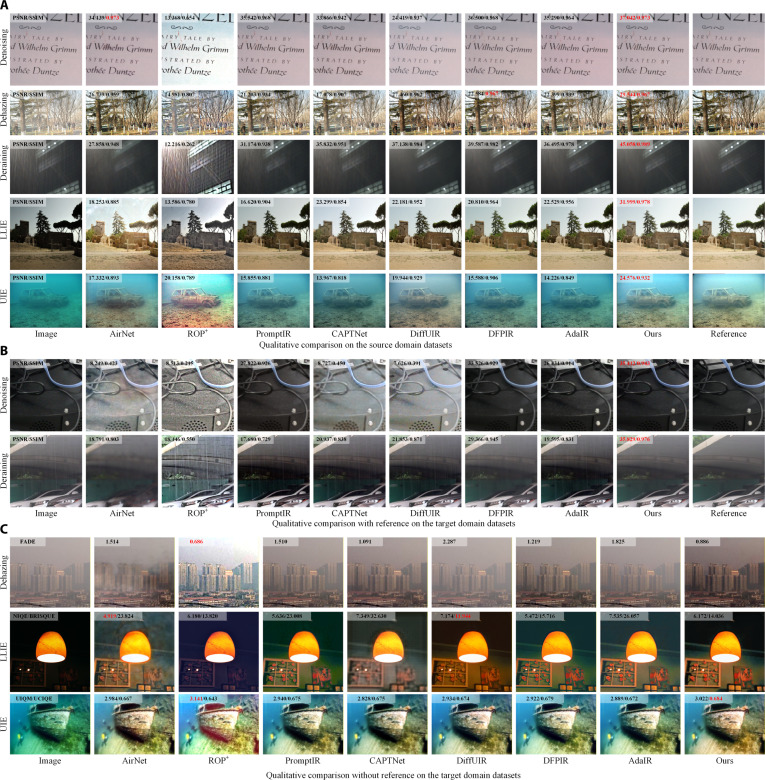
Visual comparisons of restored results on the source and target domain datasets. (A) Qualitative comparison on the source domain datasets. (B) Qualitative comparison with reference on the target domain dataset. (C) Qualitative comparison without reference on the target domain dataset.

Although objective metrics can provide desired performance comparisons, the perceptual experiences of human vision are important to be further illustrated due to the indicator bias and indirect evaluation on nonreference datasets. Therefore, for the IR task, visualizing result images is also an indispensable way to provide a comprehensive performance comparison. To this end, Fig. 2 presents the visual comparison of restored results from different methods on the source and target domains: (a) reference-based results for all 5 tasks on the source domain datasets; (b) reference-based results for denoising and deraining tasks on the target domain datasets; and (c) nonreference results for dehazing, LLIE, and UIE tasks on the target domain datasets. In addition, Fig. S2 presents a detailed comparison on the source domain in the Supplementary Materials for the performance of different models in restoring image details. In general, the following conclusions can be obtained from the results:

1. As shown in Fig. 2A, the proposed model can recover clean images visually similar to their reference counterparts, i.e., desired pixel similarity to remove degradations and perceptual similarity to recover textures and details, such as that in the objective evaluation. Considering that the proposed model can produce visual-pleasant images across different degradation patterns and data domains, the visualization results also support the technical modules in this work, i.e., degradation pattern identification and domain adaptation. To be specific, in the denoising task, the proposed model suppresses noise without introducing additional artifacts and finally obtains a leading PSNR score. For the dehazing task, most baselines suffer from residual haze and blurred edge textures, represented by inferior performance in both pixel-level and structural similarity metrics compared to the proposed model. The proposed model achieves the most substantial performance improvements on the deraining task due to the strong ability to remove rain streaks while preserving fine details, such as that in the objective evaluation. DFPIR also demonstrates a promising performance for removing rain streaks in the objective results. Although DiffUIR can also remove rain streaks, the diffusion-based paradigm tends to generate smooth textures to the loss of fine details, resulting in lower PSNR and SSIM. In the LLIE task, the proposed model enhances brightness in a balanced manner while restoring dark region details with high fidelity. For the UIE task, the proposed model effectively corrects the blue–green color cast typical in underwater scenes.
2. In real-world scenarios (target domain), as shown in Fig. 2B and C, the result image visualizations are also impacted by unseen degradations from the distribution shifts, mainly concerning the diversity and complexity of real-world scenarios. In the denoising task, almost all baselines misidentify the dark objects and finally regard them as low-light regions to perform the LLIE task, thereby performing excessive brightness correction to further introduce additional noise and artifacts. Notably, although obtaining the correct degradation patterns, DFPIR suffers from substantial loss of texture details. Meanwhile, PromptIR and AdaIR can enhance contrast in certain regions but still exhibit limited noise suppression. For the dehazing and deraining tasks, the domain differences provide extra challenges in removing haze and rain, and most baselines struggle to produce desired restoration with an unpleasant visual experience. In the LLIE task, due to the high task and technical challenges, artifacts, blurring, and color distortion are commonly induced among the comparative methods, which indicates the generalization and robustness limitations caused by training only on closed scenario data without explicit adaptation mechanisms. In contrast, the proposed model can effectively mitigate the distribution shifts through CSCL and domain adaptation, substantially improving degradation pattern identification to support dedicated restoration tasks.
3. As can be seen from the results, ROP^+^ fails to obtain expected visual-pleasant clean images on both the source and target domain datasets. In general, ROP^+^ struggles to suppress noise in most tasks (such as deraining) and often introduces visible color distortions, as indicated by the low PSNR and SSIM for reference metrics. Even for the highest nonreference metrics, the ROP^+^ also suffers from a poor visualization experience, which indicates the bias and sensitivity of the evaluation metrics, and also supports the combined objective metrics and subjective visual experience for AiOIR tasks. Fortunately, by combining objective and subjective evaluation, the proposed model can obtain the desired performance for different image enhancement tasks across different data domains, providing higher generalizability and applicability in real-world scenes.

### Ablation study

#### Degradation aware and analysis

An ablation study is conducted to validate the degradation awareness and analysis within the source domain. Table 1C reports the quantitative performance of several model variants on the source domain datasets4. Specifically, the baseline is only with the encoder and decoder architecture, without the proposed DAAM module or any additional strategies. It is also noted that both CSCL and the domain adaptation strategy are correlated with the DAAM. The results show that the 2 core components of the DAAM provide complementary and essential contributions to overall performance improvements. Specifically, the baseline includes only an encoder and decoder, without the proposed DAAM module or any additional strategies. Notably, both CSCL and the domain adaptation strategy depend on the presence of the DAAM.

**Fig. 3.**
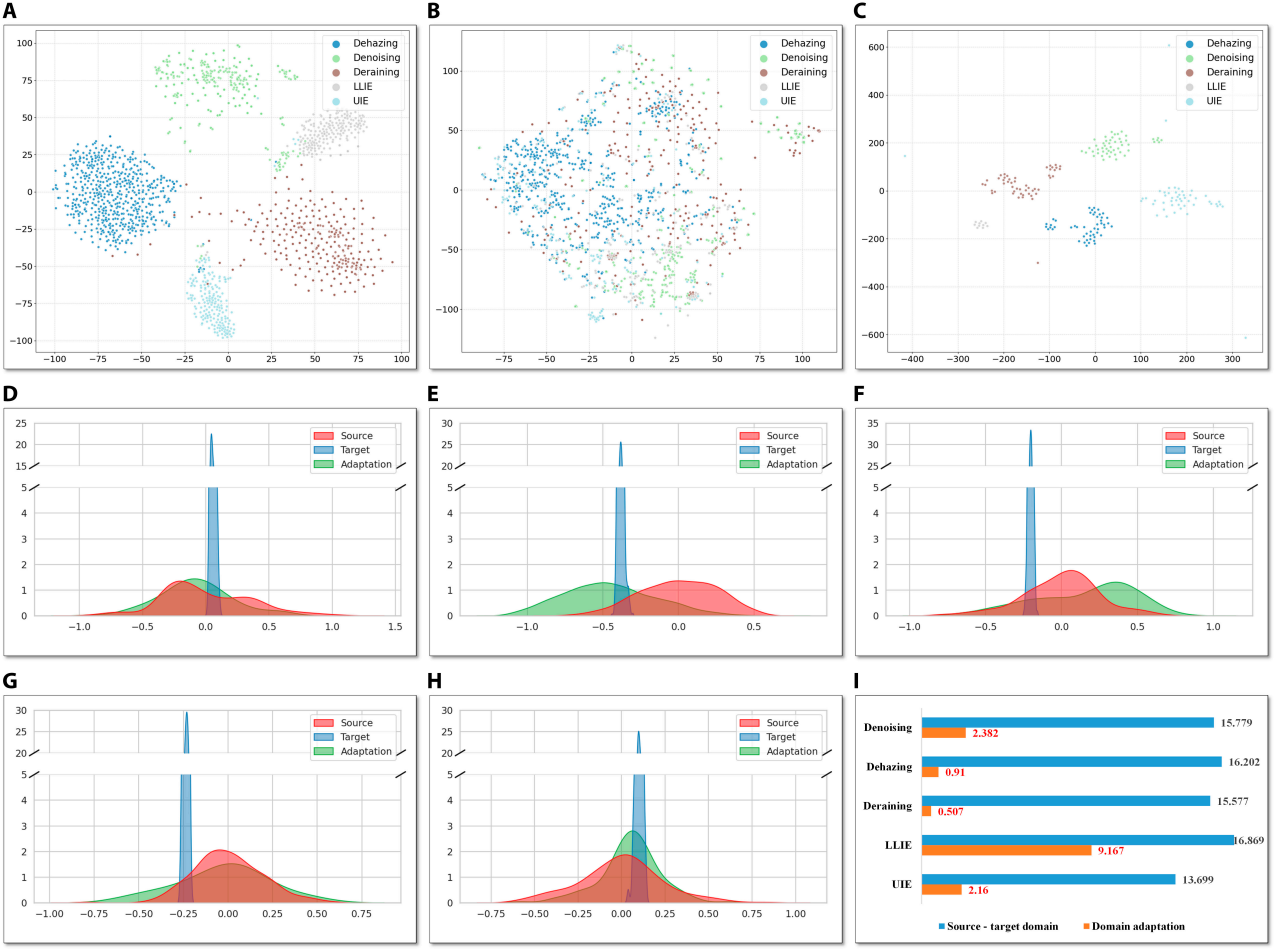
(A) and (B) are t-SNE visualizations of degradation features of w/ CSCL and w/o CSCL on the source domain datasets. (C) is the t-SNE visualization of degradation features on the target domain datasets (choosing 50 samples randomly in denoising, dehazing, deraining, and UIE tasks due to severe sample imbalance across datasets). (D) to (H) show the density plot analysis for denoising, dehazing, deraining, low-light image enhancement (LLIE), and underwater image enhancement (UIE) tasks, respectively. In the plots, source represents the density of the source domain, target represents the density of the raw target domain, and adaptation represents the density of the target domain after adaptation. The more similarity of source and adaptation densities indicates the effectiveness and efficiency of the knowledge transferability and prototype matching in handling distribution shifts. (I) is Kullback–Leibler (KL) divergence for source-target and source-adaptation in all 5 tasks; the smaller value indicates a better similarity.

CSCL: It can be seen that introducing CSCL achieves a great performance improvement across most tasks, which captures both shared and distinctive features across samples under different degradation patterns to formulate a more separable representation space. The features optimized by CSCL can guide the backbone to more effectively and precisely remove noise, haze, rain streaks, and other degradations. To further validate the DAAM in identifying the degradation patterns, t-distributed Stochastic Neighbor Embedding (t-SNE) is applied to project the degradation features from the DAAM into a 2-dimensional space, as shown in Fig. 3A and B. With CSCL, the degradation patterns are formulated as clear clusters in a compact and distinct manner, with reduced intraclass distances and increased interclass separations. As the model, without CSCL, degradation features corresponding to different patterns are heavily correlated without any clear clusters, indicating a limited ability to distinguish distinct degradation patterns. The feature clusters demonstrate the pivotal role of CSCL in identifying the different degradation categories, which is the key motivation to enhance the AiOIR task. Finally, the separable and informative representations enable the CSCL of the DAAM to perceive and distinguish multiple degradations without reference images, thereby providing an effective supervisory signal for IR.

Codebook: (a) As can be seen from the results, the proposed model without the codebook obtains inferior performance for all tasks, which validates the representation ability to the degradation patterns in a cross-domain latent space. Compared to CSCL, the w/o codebook obtains the lowest performance metrics without informative feature representations, which fails to provide high-quality priors across tasks to enhance robust and generalizable representations for diverse degradations. By combining them, the full model outperforms selective baselines, achieving the highest evaluation scores across most tasks in the source domain datasets, which validates the proposed model with enhanced degradation pattern identification to perform IR. (b) Meanwhile, the size of the codebook plays a pivotal role in representing degradation patterns, balancing representational capacity and model stability. To analyze the impact of this hyperparameter, experiments evaluate different codebook sizes: 256, 512, and 1,024, as shown in Table 2. The results show that reducing the size to 256 leads to a noticeable performance drop (approximately 10.7% in PSNR), which indicates that a smaller codebook is insufficient to capture the diversity of degradation patterns and fails to form discriminative degradation prototypes. In contrast, increasing the codebook size to 1,024 also results in a decrease of performance (approximately 4.3% in PSNR), where an oversized codebook may introduce redundant representations, i.e., learning nongeneralizable features or noise. In summary, an appropriate codebook size facilitates the formation of a stable and effective degradation representation space.

**Table 2.** Objective performance comparison of different variants in ablation study, where the best (second-best) performance is indicated in boldface (italics); ↑ indicates that higher values are better, and ↓ indicates that lower values are better

(A) Ablation study on the source domain											
Variant	Venue	Denoising		Dehazing		Deraining		LLIE		UIE	
		SSIM↑	PSNR↑	SSIM↑	PSNR↑	SSIM↑	PSNR↑	SSIM↑	PSNR↑	SSIM↑	PSNR↑
Size = 256	Codebook	0.889	36.558	0.882	26.655	0.855	29.856	0.777	19.338	0.794	20.580
Size = 1,024		0.947	39.742	0.928	29.120	0.913	32.181	0.836	20.327	*0.829*	*21.078*
ResNet	Backbone	0.936	38.397	*0.936*	28.775	*0.914*	*32.210*	*0.847*	20.612	0.825	21.069
MetaFormer		*0.950*	*39.824*	*0.936*	*29.198*	0.910	32.050	0.843	*20.803*	0.825	21.040
β = 0.1	Loss	0.750	31.669	0.885	23.993	0.746	25.166	0.765	18.764	0.790	19.585
β = 0.4		0.930	39.028	0.920	28.885	0.901	32.007	0.832	20.747	0.816	20.756
Optimal model	Ours	**0.952**	**40.101**	**0.938**	**29.715**	**0.945**	**35.193**	**0.872**	**22.671**	**0.833**	**21.208**
(B) Ablation study on the target domain											
Variant	Venue	Denoising		Dehazing		Deraining		LLIE		UIE	
		SSIM↑	PSNR↑	FADE↓		SSIM↑	PSNR↑	NIQE↓	BRISQUE↓	UIQM↑	UCIQE↑
Size = 256	Codebook	0.864	25.298	1.879		0.837	21.705	5.212	15.894	2.541	0.614
Size = 1,024		0.872	*26.420*	*1.620*		*0.843*	*23.719*	5.142	14.418	2.632	0.615
ResNet	Backbone	0.851	25.323	1.864		0.842	22.602	5.487	14.632	2.651	0.568
MetaFormer		0.863	26.010	1.683		0.841	22.663	5.101	14.554	2.714	0.611
β = 0.1	Loss	0.738	19.753	1.942		0.780	21.023	5.748	24.008	2.520	0.590
β = 0.4		*0.880*	26.196	1.708		0.830	22.067	5.045	15.535	2.695	*0.620*
TENT	TTA	0.838	23.074	1.667		0.823	20.973	4.961	14.957	2.675	*0.620*
SAR		0.862	25.253	1.677		0.834	21.676	*4.946*	*14.074*	*2.727*	0.617
Optimal model	Ours	**0.883**	**27.204**	**1.585**		**0.854**	**23.765**	**4.814**	**13.450**	**2.811**	**0.626**

LLIE, low-light image enhancement; UIE, underwater image enhancement; TTA, Test-Time Adaptation

**Fig. 4.**
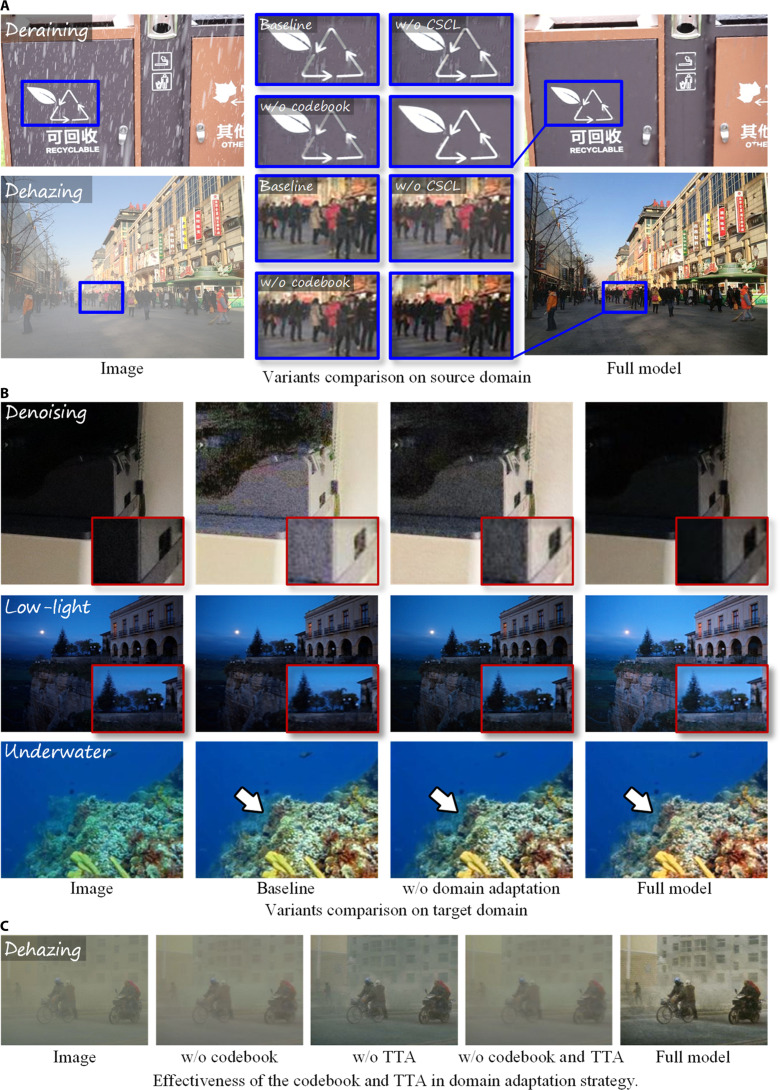
Comparisons of different variants in the ablation study. (A) Variants comparison on source domain. (B) Variants comparison on target domain. (C) Effectiveness of the codebook and TTA in domain adaptation strategy.

In addition, to further provide a comprehensive comparison, the samples of deraining and dehazing tasks are selected to perform the visualization in Fig. 4A. In general, the visualization results are the same as that of the objective results; as the blue marked regions, CSCL and the codebook achieve similar results for IR compared to the baseline model, and the best visual-pleasant images are produced by the full model.

#### Domain adaptation

Except for the ablation experiments on the source domain, ablation studies are also performed to validate the domain adaptation strategy on the target domain. These experiments aim to analyze the specific contribution of the proposed domain adaptation approach within a cross-domain setting. Table 1D summarizes the objective evaluation results for the different model variants on the target domain datasets, while Fig. 4B visualizes the resulting images to provide subjective evaluation. The model variants concern CSCL, the codebook, and TTA, in which the cross-domain latent representations for degradation features serve as the bridge between source and target domain datasets. Specifically, the configuration of the w/o codebook removes the static codebook and retains the online TTA mechanism, which is designed to examine the TTA performance without well-structured latent space (i.e., the codebook) for domain adaptation. The configuration of the w/o TTA keeps the full static codebook trained with CSCL but disables online TTA refinement during the testing phase, which separates and quantifies the performance of static cross-domain matching via the codebook. The configuration of the w/o domain adaptation removes both the codebook and the TTA components, serving as a baseline without any domain adaptation mechanisms.

The reported results demonstrate that w/o CSCL and w/o TTA obtain relatively lower performance reduction, which can be attributed to the fact that (a) CSCL enhances the model performance in the source domain datasets with limited impacts on the target domain datasets, and (b) although TTA is dedicatedly designed for domain adaptation, the implementations without informative degradation features fail to transfer the learned knowledges from source to target domain datasets, thereby supporting the motivation of the domain adaptation with the proposed TTA strategy. Furthermore, the w/o TTA obtains better performance than that of the w/o CSCL for the denoising, dehazing, and deraining tasks, which validates the required domain adaptation in the target domain datasets. As to the high technical challenges in LLIE and UIE tasks, the w/o CSCL obtains inferior performance than that of the w/o TTA, indicating an important role in learning shared intrinsic features of degradation patterns to formulate cross-domain latent space and further enhance the degradation pattern identification. In addition, the w/o codebook suffers from a larger performance gap across different tasks, which validates its ability in the knowledge transferability between the source and target domain datasets. Finally, by removing both the codebook and the TTA, the proposed model only achieves the worst performance (only comparable with the baseline), indicating that the designed domain adaptation strategy (i.e., online fine-tuning based on the well-learned cross-domain representation of degradation patterns) plays a crucial role in bridging the statistical gap between source and target domains.

In summary, the codebook maps target features into the latent feature space learned from the source domain, based on which TTA refines the model with respect to target statistics in an online manner. CSCL ensures that degradation patterns can be well identified to provide a solid foundation between the source and target domains. The full model with the 3 components has the ability to achieve higher-quality IR on unseen target data. In addition, Fig. 3C presents the dimensionality-reduced visualization of degradation features on the target-domain dataset. Benefiting from the proposed domain adaptation strategy, different degradation patterns form clear clusters, which provide effective guidance for the model to restore images with substantially diverse data distributions.

Similar to the ablation experiments in the source domain, Fig. 4B presents the visual improvements obtained by the domain adaptation strategy, in which the proposed domain adaptation strategy (full model) achieves a notable visual improvement, i.e., more balanced and natural restoration performance across various degradation patterns in the target domain. To be specific, in the denoising task, the baseline and the w/o domain adaptation variants fail to correctly identify degradation patterns, resulting in considerable deviation over reference images, i.e., residual noise, spurious artifacts, blurred surface textures, and diminished edge sharpness. In the LLIE task, the baseline and the w/o domain adaptation variant provide only limited brightness enhancement at the expense of losing substantial details in the distant scene, while the proposed model achieves balanced global brightness with retained fine textures and natural color contrast. In the UIE task, the baseline and the w/o domain adaptation variants fail to correct pervasive color cast and deliver low contrast, which obscures coral structures and marine details, while the proposed model recovers subtle underwater textures with enhanced scene contrast.

By confirming the domain adaptation, Fig. 4C further illustrates the resulting contributions of the codebook and TTA within the domain adaptation strategy. As can be seen from the results, the w/o domain adaptation presents almost identical haze with the original noisy image: extreme low contrast, obscured building outlines. The w/o TTA obtains a better visual experience than that of the w/o codebook, which can be attributed to the fact that the informative cross-domain representation space for degradation features is the key bridge to perform the domain adaptation. Without the codebook, only TTA still fails to achieve the desired restoration performance, and their combination finally harvests the best performance to validate the proposed domain adaptation strategy, as well as the motivation to address the distribution shifts among data domains.

#### Backbone architecture

To assess the impact of different backbones on restoration performance, 3 representative backbone architectures are compared: ViT, the default Transformer-based backbone employed in the proposed model; ResNet, a classical Convolutional Neural Network (CNN) architecture; and MetaFormer, a hybrid design integrating CNN and Transformer components. In the experiments, all other modules remain identical with only the backbone substituted, and the results are presented in Table 2.

The results show that the proposed framework (with ViT backbone) exhibits strong adaptability across different backbones, obtaining the most balanced performance for both restoration accuracy and computational efficiency. Although ResNet offers a slightly faster inference time (0.036 s on the source domain and 0.249 s on the target domain), it suffers from a noticeable drop in restoration quality and exhibits a higher parameter count (15.41 M) and Floating Point Operations (FLOPs, 227.72 G). In contrast, the MetaFormer achieves a relatively higher performance (close to ViT) but incurs a substantially higher computational overhead in parameter count (29.98 M), FLOPs (482.57 G), and inference time (0.16 s on the source domain and 0.349 s on the target domain).

From the task perspective, the IR highly relies on capturing long-range dependencies and integrating nonlocal contextual cues. In this context, the proposed model with the ViT backbone has the ability to extract flexible features from degraded input images via global self-attention mechanisms, and finally yielding richer representations for subsequent fusion with degradation features to generate clean images. In comparison, CNN-based backbones are constrained by static local receptive fields, and hybrid architectures offer limited global modeling capability. In summary, from the task requirements and experimental results, the ViT is selected as the backbone of the proposed model.

### Feature distribution analysis with similar patterns

To further validate the proposed model in achieving the AiOIR task from controlled scenarios to the real world, the feature distributions of images from the source domain (source), the raw target domain (target), and the target domain after adaptation (adaptation) are analyzed in Fig. 3 using density analysis (Fig. 3D to H) and KL divergence comparison (Fig. 3I) for data from different domains.

In the density plots, the principal component analysis is performed to reduce high-dimensional vectors to their leading principal components, and one-dimensional projections of these components are applied to implement kernel density estimation. In general, the density has extreme clear differences between the source and target domains, which indicates the distribution shifts among different datasets and supports the motivation to achieve the AiOIR task from controlled scenarios to the real world. By performing the proposed domain adaptation strategy, the density similarities of all tasks are highly enhanced by fine-tuning the learned codebook prototypes, which demonstrates the effectiveness and efficiency of the knowledge transferability and prototype matching in handling distribution shifts, and finally enhances the final objective and subjective performance.

In addition, the KL divergence for source-domain and source-adaptation is also quantified to provide a comprehensive comparison, in which a smaller KL value indicates a better similarity. In this experiment, the full-dimensional degradation features can be applied to measure KL divergence to capture all components of the learned representations. As shown in Fig. 3I, the results show that the huge gaps of the KL divergence for different tasks provide the quantitative comparison for the density analysis, in which the gaps for LLIE and UIE tasks are relatively smaller than that of other tasks due to the aforementioned task challenges. In summary, the in-depth analysis for the feature distribution under similar degradation patterns validates the prototype matching from learned representations in the source domain to the target domain, thereby providing more reliable and stable priors for the subsequent restoration branch in the proposed model.

### Efficiency analysis

#### Computational complexity and inference time

**Fig. 5.**
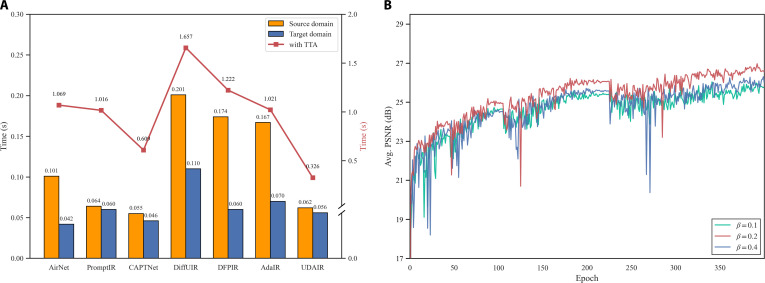
(A) Average inference time of different methods on the source and target domain datasets. The bar chart corresponds to the left *y*-axis (black labels), while the red curve corresponds to the right *y*-axis (red labels). (B) Visualization of loss curves during model training, where the *y*-axis represents the average PSNR evaluated on the source domain.

To comprehensively assess the efficiency of the proposed model, the static model complexity (including parameter count and FLOPs) and dynamic inference time with TTA enabled are considered as metrics. As shown in Fig. 5A and Fig. S4, the proposed model demonstrates clear advantages in both static complexity and inference speed. The model not only maintains a relatively small number of parameters and FLOPs but also achieves an average inference time of 0.062 s in the source domain, indicating the efficiency of the network architecture. However, substantial domain shifts occur between closed and real-world scenarios, making TTA essential for maintaining robustness and restoration quality. To evaluate the computational complexity with the TTA module, average inference times in the target domain are further measured across all methods. The results demonstrate the superior efficiency of the proposed TTA strategy, achieving an average inference time of only 0.326 s. In contrast, the second-best method, CAPTNet, with the Test entropy minimization (TENT) requires 0.609 s, while other comparative methods suffer from 3 to 5 times higher computational overhead. In general, the proposed model maintains competitive computational performance in terms of FLOPs and parameter count, as well as the efficient TTA strategy, thereby enhancing the feasibility of deploying TTA-based approaches in practical systems.

While the TTA mechanism inevitably introduces additional latency compared to static inference, it is essential for bridging distribution shifts since pretrained static models frequently fail to generalize in unseen real-world scenarios. Therefore, considering the prohibitive costs associated with collecting pixel-aligned data for offline retraining, the incurred inference time represents a viable solution for ensuring immediate model availability in data-scarce scenarios.

#### Convergence efficiency via weighted loss

To achieve robust training procedure, the proposed framework is optimized via a composite objective function, including a weighted Mean Absolute Error (MAE) and a CSCL loss. In this formulation, $L_{\mathrm{MAE}}$ drives the model to learn fundamental IR capabilities, and $L_{\text{CSCL}}$ serves as an auxiliary term for the unsupervised extraction of degradation pattern features. Given the dominant role of $L_{\mathrm{MAE}}$ in restoration tasks, its weight coefficient α is fixed to 1. Therefore, the key objective of parameter selection lies in determining an appropriate value for the hyperparameter β, which balances pixel-level reconstruction and degradation pattern identification.

To this end, a sensitivity analysis is conducted on β to examine model convergence and final performance under $\beta \in \left\{0.1, 0.2, 0.4\right\}$. The average PSNRs during training for different settings are illustrated in Fig. 5B. When β=0.1, the training process remains relatively stable, with only an inferior final PSNR, indicating that the contrastive learning module does not fully exploit its potential in degradation pattern discrimination. When β=0.4, the large weight of the auxiliary contrastive loss introduces considerable training instability, as well as degraded performance. Fortunately, the proposed model with β=0.2 achieves the most stable convergence and the highest average PSNR throughout the training process, which finally serves as the optimal options for model training.

### TTA analysis

To further evaluate the effectiveness of the proposed TTA strategy in this work, additional experiments are conducted by integrating several representative TTA methods, including TENT [27], which minimizes prediction entropy to increase confidence on target domain samples, and Sharpness-aware and Reliable entropy minimization (SAR) [43], which employs sharpness-aware updates to guide optimization toward flat minima, thereby improving stability during TTA.

Comprehensive evaluations are performed on UDAIR, UDAIR-TENT (replacing the proposed TTA with TENT), and UDAIR-SAR (replacing the proposed TTA with SAR) across all target domain tasks, as shown in Table 2. The results show that the proposed UDAIR consistently surpasses both TENT- and SAR-enhanced variants on almost all tasks and metrics, which finally validates the domain adaptation strategy (DAM and TTA) in this work. Meanwhile, considering the runtime during the TTA phase: the proposed UDAIR, UDAIR-TENT, and UDAIR-SAR require an average of 0.326, 0.694, and 1.185 inference time, respectively.

In addition, generic TTA strategies (i.e., TENT) are implemented across all baseline models to evaluate potential performance gains. Nevertheless, only negligible improvements or erratic performance fluctuations are observed relative to the static counterparts, as comprehensively analyzed in the “Limitations of Generic TTA on Image Restoration” section in the Supplementary Materials.

In summary, by designing the DAM and restoration-specific TTA strategy, the proposed model harvests higher performance and efficiency, which validates the core contributions in addressing the key cross-domain adaptation challenges for AiOIR tasks.

### Statistical significance analysis

To evaluate the reliability of the proposed model, the Wilcoxon signed-rank test (W-test) is adopted for statistical analysis to eliminate the influence of random errors.

The W-test between the proposed model, baseline variants, and the State-of-the-Art (SOTA) method (AdaIR) are systematically compared across 5 tasks, as shown in Table 3. AdaIR is selected as the primary comparison method for this experiment because the most robust comprehensive performance among all existing methods is demonstrated by this approach. Specifically, using mean-rank strategy across all 19 metrics, AdaIR achieves a mean rank of approximately 3.16, second only to the proposed model with a mean rank of approximately 1.68. This selection ensures that statistical significance is measured against the most competitive SOTA alternative. Experimental results demonstrate that, in more than 90% of the test cases, the resulting P values are smaller than 0.05, indicating statistically significant differences. Furthermore, for approximately 70% of the comparisons, the P values are less than 0.001, demonstrating robust statistical significance. Meanwhile, the P values on the UIE task suffer from higher value due to the inferior performance evaluation metrics on UDAIR-AdaIR. In summary, the experimental results provide robust statistical evidence that the proposed model consistently achieves statistically significant performance improvements across diverse and complex restoration environments.

**Table 3.** Wilcoxon signed-rank test results for statistical significance on the source domain (A) and the target domain (B). The P values assess the significance of the proposed model compared against the baseline (the base variant in Table 1C and D) and AdaIR, where P < 0.05 is considered as a significant improvement.

(A) P values on the source domain										
Comparison method	Denoising		Dehazing		Deraining		LLIE		UIE	
	SSIM	PSNR	SSIM	PSNR	SSIM	PSNR	SSIM	PSNR	SSIM	PSNR
Baseline	<0.001	<0.001	<0.001	<0.001	<0.001	<0.001	<0.001	<0.001	<0.001	<0.001
AdaIR	<0.001	<0.001	<0.001	<0.001	<0.001	<0.001	<0.001	<0.001	0.014	0.964
(B) P values on the target domain										
Comparison method	Denoising		Dehazing		Deraining		LLIE		UIE	
	SSIM	PSNR	FADE		SSIM	PSNR	NIQE	BRISQUE	UIQM	UCIQE
Baseline	<0.001	<0.001	<0.001		<0.001	<0.001	0.013	0.024	<0.001	<0.001
AdaIR	0.001	0.005	<0.001		<0.001	<0.001	0.032	0.032	<0.001	<0.001

LLIE, low-light image enhancement; UIE, underwater image enhancement

## Discussion

This work investigates the discrepancy in feature distributions across domains, which remains the primary factor that limits the performance of AiOIR models in diverse real-world scenarios, and further constrains the practical deployment of these models. In this work, a UDAIR computer vision model is proposed to address 2 core challenges: handling multiple complex degradation patterns via a unified model architecture, and aligning source-target domain feature distributions through a domain adaptation strategy. The DAAM is proposed to employ CSCL to learn the intrinsic features of degradation patterns without relying on reference images. Within the DAAM, the codebook constructs a cross-domain latent space to store degradation pattern prototypes by learning shared intrinsic priors of degradation patterns, effectively mitigating the impact of performance decline in degradation pattern identification caused by cross-domain distribution shifts. A CSCL strategy prevents the model from focusing on sample-wise local details that are irrelevant to degradation, which also serves as the key to bridge the domain gap. In this way, the model can effectively leverage the knowledge and priors learned from the source domain, alleviating the performance decline in degradation pattern identification caused by distribution shifts. During TTA, the DAM is dynamically activated to fine-tune the target features through online optimization, thereby minimizing distributional inconsistency. Notably, TTA and the DAM are activated exclusively during target domain inference and remain inactive during other cases. This dual mechanism enables the model to effectively leverage source domain degradation priors while adaptively accommodating emerging target domain features, thereby establishing a cross-domain IR framework with enhanced robustness and generalization capability. Consequently, the proposed model overcomes the limitations of relying solely on synthetic or low-diversity datasets and demonstrates strong generalization in real-world scenarios. More importantly, the experimental results highlight the essential roles of the domain adaptation strategy, the codebook, and the TTA mechanism in generalizing models trained in closed and controlled scenarios to real-world scenarios without introducing additional real-world samples during training. It is believed that this work provides a solid foundation for exploring more effective techniques for real-world applications in the future.

### Limitations

Although the UDAIR demonstrates substantial performance improvement in multiple degradation tasks, it is also found that degradation identification is also confused for a certain degradation pattern with different intensities (as shown in Fig. S5). For example, although compacted feature clusters can be formulated by different restoration tasks, the synthetic Berkeley Segmentation Dataset (BSD) [44] dataset under Gaussian noise with σ=15,25,50 is also distinguished to formulate different clusters. It can be attributed to the fact that the energy distribution and texture statistics of the degraded images are markedly changed as σ increases from 15 to 50, which further disables the DAAM to capture informative representations to identify the degradation patterns. In addition, for the UIE task, the proposed model only obtains suboptimal performance on the source domain with signification improvements on the target domain due to the different degradation patterns with other tasks (coupled impact factors with scattering, light absorption, etc.), which also supports the motivation of this work to identify the degradation patterns.

### Applications

The proposed model shows strong applicability in real-world scenarios with diverse degradations. In airport surveillance, where haze, rain, and low-light conditions commonly occur, the proposed model provides an all-in-one solution that restores clear images using a unified architecture while being optimized to achieve real-time processing speeds ranging from 25 to 60 frames per second (FPS) on modern GPUs with minimal impact on restoration quality, as illustrated in the “Application Scenarios in Airport Surveillance System” and “Efficiency Optimization for Airport Surveillance Systems” sections in the Supplementary Materials. Beyond airport surveillance, the model can be adapted for safety-critical monitoring systems, autonomous driving, UAV, and other applications requiring reliable visual perception under complex environmental conditions.

### Future directions

In this work, the UDAIR achieves the AiOIR task by automatically identifying and restoring single degradation patterns. As analyzed in the Roadmap (see the “All-in-One Image Restoration Task Overview and Roadmap” section in the Supplementary Materials), the evolution of the field has progressed from SDIR to MDIR, and finally to the current AiOIR paradigm. While AiOIR offers substantial advantages by using unified parameters without task-specific priors, its current efficacy in practical scenarios with coupled degradations (such as simultaneous rain, haze, and low-light conditions) is fundamentally constrained by the prevalent single-degradation training paradigm. In this context, as the key to data-driven methods, it is highly required to construct multidegradation benchmark datasets to simulate real-world scenes, i.e., each sample has multiple degradation patterns. Meanwhile, based on the new dataset, the AiOIR methods can be studied to simultaneously recognize heterogeneous degradations and holistically restore clear images through unified frameworks, which play a crucial role in advancing AiOIR research for practical real-world vision systems under complex environmental conditions.

## Methods

### Framework

#### Motivation

For real-world scenes, the images are generally with diverse and complex degradation, and existing AiOIR methods are usually trained on closed and controlled datasets. Due to feature distribution shifts between the source and target domains in real-world applications, the IR models suffer from limited generalization on the test environment (i.e., target domain). Specifically, distribution shifts impact the applicability of directly transferring the learned representations from source to target domain data, resulting in a substantial performance decline and poor perceptual experience. Moreover, real-world samples are often variable and unpredictable, which greatly increases the difficulty of domain adaptation (e.g., some TTA methods require simultaneously aligning the feature spaces between the source and target domains to achieve domain adaptation). Therefore, in this work, samples from the target domain are only visible during inference.

#### Solution

**Fig. 6.**
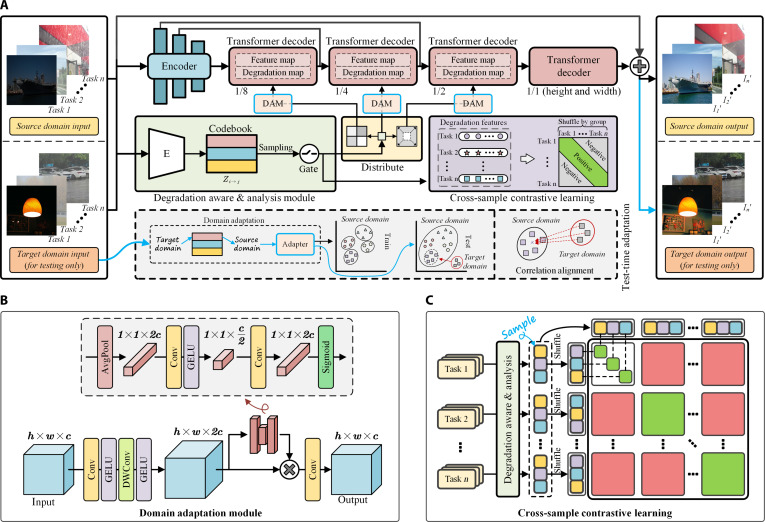
(A) Overview of the proposed framework. The blue pipeline represents the dynamic domain adaptation strategy activated exclusively during target domain inference. (B) Schematic diagram of the Domain Adaptation Module. (C) Schematic diagram of the Cross-Sample Contrastive Learning.

To address the mentioned challenges, a UDAIR framework is proposed to effectively leverage the knowledge learned from the source domain to the target domain, as shown in Fig. 6A. In IR tasks under unseen distributions of degradation patterns, the key of the proposed model depends on accurately identifying degradation patterns and capturing degradation features. To this end, a codebook-based DAAM is proposed for perceiving and analyzing degradation patterns. The codebook, as the core component of the DAAM, stores degradation prototypes with shared intrinsic priors learned from the source domain, which are optimized under the guidance of CSCL. In addition, a CNN-based feature extractor is designed before the codebook to extract degradation features from the input image. Following the codebook, a gating mechanism is applied to filter and weight the features, thereby selecting the most representative information. Overall, the design of the DAAM is as follows:

$$\begin{array}{c} \begin{array}{c} f_{d}=\text{Extractor}\left(I\right),\\ f_{c}=\text{codebook}\left(f_{d}\right),\\ F_{d}=\text{gate}\left(f_{c}\right), \end{array} \end{array}$$ (6)

where I is the input degraded image; $f_{d}$ denotes the continuous features extracted by the CNN-based feature extractor; $f_{c}$ is the quantized representation obtained from the codebook; and $F_{d}$ represents the output of the gating mechanism, which serves as the final output of the DAAM. After scale alignment, the degradation features $F_{d}$ are propagated into the decoder to guide the backbone for the restoration of a clear image:

$$\begin{array}{c} \begin{array}{c} F_{enc}=\text{Encoder}\left(I\right),\\ F_{dec}=\text{Decoder}\left(\text{Concat}\left(F_{d},\;F_{enc}\right)\right),\\ \;\hat{I}=\text{Conv}\left(F_{dec}\right)+I, \end{array} \end{array}$$ (7)

where Concat is the concatenate operation; Conv is the convolution operation to restore the dimensions to the original image size; $\hat{I}$ is the restored clear image; and Encoder and Decoder represent the encoder and decoder, respectively.

For the perspective of cross-domain degradation, a CSCL mechanism is proposed to guide models to capture and learn the shared intrinsic features of degradation patterns. Specifically, the extracted image components are grouped by different restoration tasks, and a random permutation is applied to generate positive sample pairs that share the same semantics but exhibit different degradation patterns. Through CSCL, the IR models are encouraged to autonomously capture and analyze the representation of degradation features without relying on references or labels. Compared to the patch-level positive pairs from different regions within the same image, CSCL has the ability to cover diverse representations of a certain degradation pattern from different samples, allows the model to better capture the commonalities of degradation features, focuses on the essential differences among degradation patterns, and ultimately improves its generalization ability across various degradation scenarios.

A domain adaptation strategy is proposed to address data distribution shifts via a codebook and TTA mechanism. The codebook establishes a cross-domain latent space to represent degradation pattern prototypes, enabling the model to leverage learned knowledge and priors from the source domain. The domain adaptation is implemented by matching the prototype representations in the codebook for source and target domain data. Since the data distribution of each sample in real-world scenarios may differ substantially, TTA dynamically re-fine-tunes the model parameters based on test images in a sample-level manner to further reduce the domain discrepancies caused by inherent discretization errors and information loss during prototype matching, thereby alleviating generalization errors resulting from interdomain distribution shifts. Specifically, a DAM is proposed to dynamically update the model weights for each sample during the TTA procedure. To enhance the representational capacity, the cluster center of the learned features for each degradation pattern in the source domain is designated as the anchor point, since it is regarded as a more general and universal representation. By guiding target samples toward these anchor points, the DAM effectively reduces the impact of cross-domain mapping errors caused by sample-specific distribution variations. During testing on target domain samples, the procedure is as follows:

$$\begin{array}{c} \begin{array}{c} F_{enc}=\text{Encoder}\left(I\right),\\ F_{d}^{\prime }=T\left(\mathrm{DAM}\left(F_{d}\right)\right),\\ F_{dec}=\text{Decoder}\left(\text{Concat}\left(F_{d}^{\prime },\;F_{enc}\right)\right),\\ {\hat{I}}^{\prime }=\text{Conv}\left(F_{dec}\right)+I. \end{array} \end{array}$$ (8)

### Backbone

In the proposed model, the Vision Transformer [45] primarily serves as the core backbone for both the encoder and decoder, with each layer consisting of L Transformer blocks. Since the degradation pattern of the input image is not explicitly provided, the captured degradation features are leveraged to guide the decoder to restore images with various degradation patterns. The learned degradation features can be regarded as an abstract representation of specific degradation patterns, capturing the diverse degradations caused by complex factors in the input image through low-dimensional semantic encoding into compact feature vectors. In this framework, the backbone mainly performs the restoration task, where the encoder extracts image features, and the degradation features guide the decoder to integrate these image features for effective restoration. Specifically, before each decoder layer, the general features from the encoder layers and the degradation features from the DAAM are concatenated to provide comprehensive representations to support the IR task. It is noted that, during the TTA procedure, the DAM is further designed to align the features between the target domain and the source domain to address the distribution shifts.

The encoder is designed to extract high-dimensional features from the input image, which are then progressively downsampled to obtain low-dimensional representations. Specifically, the encoder consists of multiple Transformer blocks, each with a multihead self-attention mechanism and a feed-forward network. The input image is first divided into patches through patch embedding operation, which are then linearly projected into high-dimensional feature vectors:

$$f_{patch}=\text{PatchEmbed}\left(I\right),$$ (9)

where PatchEmbed is the patch embedding operation. These vectors are processed through the Transformer blocks to capture long-range dependencies and contextual information:

$$\begin{array}{c} \begin{array}{c} \begin{array}{c} F_{enc}^{i}={\text{Transformer}}^{\times L}\left(f_{patch}\right),\;i=1,\\ F_{enc}^{i}={\text{Transformer}}^{\times L}\left(F_{enc}^{i-1}\right),\;i=2,3,4, \end{array} \end{array} \end{array}$$ (10)

where $F_{enc}^{i}$ is the output features of each encoder layer and ${\text{Transformer}}^{\times L}$ denotes the Transformer block repeated L times (set to [4, 6, 6, 8] of each encoder layer in this work). The decoder is designed to restore the image from the low-dimensional features, which are concatenated with the degradation features to restore a clear image guided by degradation patterns. The decoder, same as the encoder, consists of multiple Transformer blocks, each with a multihead self-attention mechanism and a feed-forward network. The input features are processed through the Transformer blocks to restore the image:

$$\begin{array}{c} \begin{array}{c} \begin{array}{c} f_{dec}^{i}={\text{Transformer}}^{\times L}\left(\text{Concat}\left(F_{d},\;F_{enc}^{4}\right)\right),\;i=1,\\ f_{dec}^{i}={\text{Transformer}}^{\times L}\left(\text{Concat}\left(F_{d},\;F_{dec}^{i-1}\right)\right),\;i=2,3,\\ f_{dec}^{i}={\text{Transformer}}^{\times L}\left(F_{dec}^{i-1}\right),\;i=4,\\ F_{dec}^{i}=\text{Conv}\left(\text{Concat}\left(\mathrm{Up}\left(f_{dec}^{i}\right),\;F_{enc}^{4-i+1}\right)\right),\;i=1,2,3,4,\\ \;\hat{I}=\text{Conv}\left(F_{dec}^{4}\right)+I, \end{array} \end{array} \end{array}$$ (11)

where Up is the upsample with pixelshuffle operation; $F_{d}$ is the degradation features from the DAAM; and $\hat{I}$ is the restored image. The decoder progressively upsamples the low-dimensional degradation features to restore the original image resolution.

### Degradation aware and analysis

#### Degradation awareness

In this procedure, the convolutional neural networks serve as the feature extractor, with the primary task of progressively downsampling high-dimensional input features to extract key information to generate a low-dimensional latent representation:

$$\begin{array}{c} \begin{array}{c} f_{d}=\Phi \left(\text{Conv}\left(I\right)\right),\;\text{where}\;\Phi =\prod_{i=1}^{2}{\mathrm{Conv}}_{i}\;\circ \;\rho , \end{array} \end{array}$$ (12)

where $I\in {\mathbb{R}}^{H\times W\times 3}$ is the input image and $f_{d}\in {\mathbb{R}}^{\frac{H}{4}\times \frac{W}{4}\times 128}$ is the degradation feature; ρ denotes the ReLU activation function; and ∘ denotes the composition of functions, meaning that the output of the function on the right is used as the input of the function on the left. These degradation features are then flattened into a 2-dimensional matrix and guide the codebook to generate a set of discrete learnable representations, which can be regarded as the degradation patterns of the input image. In this work, a codebook-based degradation awareness module is proposed to quantify complex degradation patterns by a set of discrete learnable representations. These representations are defined as the degradation prototypes in this work, formulated as:

$$\begin{array}{c} f_{c}=\text{codebook}\left(f_{d}\right). \end{array}$$ (13)

Specifically, each vector at a spatial location in the low-dimensional feature $f_{d}$ is treated as an individual encoding unit and is flattened into a 2-dimensional matrix, where each row corresponds to a continuous feature vector $z_{e}$ generated by the encoder at a given spatial location. Meanwhile, the predefined codebook is an embedding matrix of size $\left[K,\;L_{v}\right]$, where K represents number of discrete codebook entries, and $L_{v}$ is the dimensionality of each embedding vector. For each flattened vector $z_{e}$, the Euclidean distance to each vector $e_{k}$ (with index k∈1,2,…,K) in the codebook is computed as:

$$\begin{array}{c} \text{Dist}\left(z_{e},\;e_{k}\right)={\left\|z_{e}-e_{k}\right\|}_{2}^{2}, \end{array}$$ (14)

where Dist denotes the Euclidean distance computation. For each $z_{e}$, a quantization operation is performed by assigning it to the discrete code indexed by $k^{*}$ with the smallest Euclidean distance, projecting the continuous feature to a discrete predefined code:

$$\begin{array}{c} k^{*}=\arg\;\min_{k}\left(\text{Dist}\left(z_{e},\;e_{k}\right)\right). \end{array}$$ (15)

After obtaining the discrete index for each $z_{e}$, the corresponding embedding vector, denoted as $z_{q}$, is retrieved from the codebook. Finally, $z_{e}$ is replaced by its nearest neighbor codebook vector:

$$\begin{array}{c} z_{q}=e_{k^{*}}. \end{array}$$ (16)

The embedding vector $z_{q}$ serves as the representation of the degradation prototype of a single image, which is reshaped into a feature map as the degradation feature $f_{c}$.

Since the gradient of the $\arg\underset{k}{\;\min}$ operation is zero almost everywhere, the Straight-Through Estimator mechanism is adopted to approximate the gradient of $z_{e}$ using $\nabla_{z_{q}}L$, which is then back-propagated into the feature extractor:

$$\begin{array}{c} \nabla_{\theta_{e}}L=\left(\nabla_{z_{e}}L\right)\cdot \frac{\partial z_{e}}{\partial \theta_{e}}\approx \left(\nabla_{z_{q}}L\right)\cdot \frac{\partial z_{e}}{\partial \theta_{e}}. \end{array}$$ (17)

This gradient signal updates the parameters of the feature extractor $\theta_{e}$, enabling future embeddings $z_{e}$ to map more accurately to the updated prototype vector $e_{k^{*}}$. Under this mechanism, the feature extractor and the codebook are driven by the same gradient source, thereby achieving coevolution and implicit alignment to achieve stable and effective training.

As the primary optimization target of the DAAM, the contrastive learning loss provides a structured semantic gradient in the latent space $z_{q}$. This gradient is simultaneously applied to the codebook prototype $e_{k^{*}}$ and the feature extractor output $z_{e}$, forcing them to converge toward a shared objective and enabling efficient implicit alignment. In the AiOIR task, the joint training is reinforced by image samples from 5 distinct degradation patterns, in which heterogeneous and diverse data distributions require the codebook to learn discriminative prototypical representations to cluster different degradations. In this context, codebook entries are actively updated and associated with the degradation prototypes corresponding to the 5 degradation categories.

As validated by Fig. 3A to C, the DAAM produces highly compact and well-separated clusters in the latent space: samples with the same degradation form tight clusters, while features from different degradations are pushed by clear cluster boundary. It is believed that the proposed model successfully learns discriminative codebook prototypes, and the feature embeddings are tightly aligned with the codebook, enabling accurate quantization of $z_{e}$ into their corresponding semantic clusters.

The designed DAAM constructs a cross-domain latent space to store prototypes capturing the shared inherent nature of degradation patterns, which effectively achieves interdomain mapping and exhibits strong cross-domain generalizability. In this context, the degradation awareness is expected to mitigate performance degradation caused by interdomain distribution discrepancies, thereby enhancing both the accuracy of degradation pattern identification and restoration performance in real-world scenarios.

Based on the learned codebook, a gating mechanism is also proposed to filter discrete features to select the most informative representations by learnable attention weights, which guide the model to focus on salient features expressing similar degradation information with high discriminability and robustness. The gating mechanism on codebook prototypes facilitates feature learning to support the subsequent contrastive learning stages:

$$\begin{array}{c} F_{d}=\text{gate}\left(f_{c}\right). \end{array}$$ (18)

Specifically, the gating mechanism is designed to adaptively weight the degradation features from the codebook; for each channel, the global embedding $E_{g}$ is obtained by:

$$\begin{array}{c} E_{g}=\lambda \sqrt{\sum_{i=1}^{H}\sum_{j=1}^{W}{\left(f_{c}^{i,j}\right)}^{2}+\epsilon }, \end{array}$$ (19)

where $f_{c}$ denotes the input feature; λ is a set of learnable scaling factor; and ϵ is a small constant to prevent division by zero. In succession, a normalization factor Z is calculated to regulate the scale of the output and stabilize the training process, enabling more effective information fusion:

$$\begin{array}{c} Z=\sqrt{\sum_{c=1}^{C}{\left(E_{g}\right)}_{c}^{2}+\epsilon }, \end{array}$$ (20)

where C is the number of channels in the feature map.

Finally, the gating coefficient g is obtained by applying a mapping function tanh to the global embedding $E_{g}$ and the normalization factor Z. The coefficient g is formulated as:

$$\begin{array}{c} g=1+\tanh\left(\gamma_{c}\cdot \frac{\sqrt{C}E_{g}}{Z}+\beta_{c}\right), \end{array}$$ (21)

where $\gamma_{c}$ and $\beta_{c}$ are learnable parameters corresponding to scaling and bias, respectively. This coefficient is multiplied (channel-wise) with the original feature to produce the final output $F_{d}$:

$$\begin{array}{c} F_{d}=f_{c}\cdot g. \end{array}$$ (22)

#### Cross-sample contrastive learning

In general, contrastive learning has the ability to automatically learn and capture degradation features for different patterns from large amounts of data samples. The core idea of contrastive learning is to tighten similar samples (with the same attribute) in an abstract high-level feature space, while enlarging the feature discrepancy for dissimilar samples (with different attributes) to enhance the feature discriminability. In the procedure of contrastive learning, the organization of the triplet sample pairs, including anchor, positive, and negative, is key to guiding the model optimization in a stable and efficient manner. In general, by determining an anchor sample, the positive sample is usually generated from the anchor sample by cropping, noising, or other augmentation operations to ensure the same degradation pattern, while negative samples are randomly obtained from images with different degradation patterns. Although existing strategies can simply organize the training samples for contrastive learning, they still suffer from undesired sample diversity and feature discriminability facing complex tasks in similar scenes.

To address this issue, in this work, a CSCL strategy is proposed to improve the positive sample pairs from the same degradation pattern, as shown in Fig. 6C. Specifically, the extracted features from samples with the same degradation patterns are grouped and randomly permuted to learn common and stable cross-sample feature representations. The detailed steps of the CSCL algorithm are presented in Algorithm S1. CSCL guides the output features $F_{g}$ to focus on the degradation-related semantic consistency to obtain the final output of the DAAM, which is regarded as having more robust and generalizable representations.

In terms of sample-pair construction, conventional contrastive learning methods typically rely on semantic consistency, class labels, or softened relational cues to define positive and negative pairs. In contrast, the core criterion of CSCL is redefined as degradation correlation. By mining the similarity of degradation features across samples that share the same degradation pattern, CSCL guides the model to focus on the common structural patterns of degradation rather than semantic correspondences. At the pairing-strategy level, a sample-level random shuffling mechanism is designed to overcome the localization bottleneck associated with traditional fixed augmentations. Unlike static augmentation pipelines, the random shuffling leverages stochastic sampling during training to dynamically generate highly diverse cross-sample combinations, which allows the training process to approximate global sample-pair interactions across the entire dataset rather than being constrained to local variations, thereby substantially improving the robustness of the learned representations.

Let the set of sample features for a certain degradation pattern d be denoted as:

Sd=s1s2…sn. (23)

where $S_{d}$ is the set of sample features for a certain degradation pattern d; and n is the number of samples.

For the set $S_{d}$ of sample features, a random permutation function π is applied to obtain a permuted order:

Sdπ=sπ1sπ2…sπn. (24)

The positive sample pairs are formulated by:

P=sisπii=1…n. (25)

The negative sample pairs are defined as:

N=sisjπsi∈Sdsjπ∈Sd′πd≠d′. (26)

where $S_{d^{\prime }}^{\pi }$ is the set of sample features for a different degradation pattern $d^{\prime }$.

Instead of considering local features from a single image, the proposed CSCL encourages the model to focus on more robust, abstract, and general semantic information on degradation patterns from different samples, thereby enhancing its generalization across different scenarios or data distributions. Combining with the random selections in batches, the constructed positive and negative sample pairs are expected to nearly cover the entire dataset to perform the model training. The resulting sample diversity in CSCL allows the model to learn only degradation-related semantic consistency between positive sample pairs, rather than the coupled features with other influential factors. The loss for CSCL is obtained by:

$$\begin{array}{c} L_{CSCL}=-\frac{1}{n}\sum_{i=1}^{n}\log\frac{\exp\left(\mathrm{sim}\left(s_{i},\;s_{\pi \left(i\right)}\right)/\tau \right)}{\sum_{D}\sum_{j=1}^{n}\exp\left(\mathrm{sim}\left(s_{i},\;s_{j}^{\pi }\right)/\tau \right)}, \end{array}$$ (27)

where sim denotes cosine similarity; τ is a tuning parameter to adjust the smoothness of the distribution; n represents the number of samples; and D denotes all degradation patterns in this work, including d and all $d^{\prime }\neq d$.

In summary, the DAAM is designed to autonomously capture and discriminate the degradation-related informative features without external references or labels, which helps address the local-feature learning to further enhance the degradation pattern identification in diverse and unseen scenarios.

### Loss function

To achieve model training, the proposed model is optimized by a weighted MAE and CSCL loss:

$$\begin{array}{c} L_{total}=\alpha L_{MAE}+\beta L_{CSCL}. \end{array}$$ (28)

where α and β are the weight coefficients for $L_{MAE}$ and $L_{CSCL}$, respectively. $L_{MAE}$ is defined as:

$$\begin{array}{c} L_{MAE}=\frac{\sum_{i=1}^{n}\left|\;f\left(x_{i}\right)-y_{i}\right|}{n}, \end{array}$$ (29)

where $f\left(x_{i}\right)$ and $y_{i}$ denote the restoration images and the corresponding reference images for the -ith sample, respectively, and n is the number of samples.

### Domain adaptation

Currently, the source domain data for the IR task is typically synthetic or captured in dedicatedly controlled scenes. However, in practical real-world applications, the target domain data usually exhibit distribution shifts of high-level features (i.e., generally different from that in the source domain), and it is hard to introduce target domain samples or distributions during training. This leads to poor generalizability when applying the model trained on source domain data to the target domain data.

To address this issue, in this work, a domain adaptation strategy is proposed to mitigate the performance degradation caused by domain distribution discrepancies. Thanks to the proposed codebook-based representations for degradation patterns, the proposed model can establish a cross-domain shared latent space to match degradation prototypes across different domains, in which the quantization errors and loss of fine-grained information are still required to be further addressed to enhance the final performance on the target domain data. To this end, in this work, the DAM and TTA are innovatively designed to achieve the feature alignment and correction between the samples of the source and target domains. Specifically, for a data sample in the target domain, a DAM is designed to achieve the feature projection from the degradation features to a predefined anchor point in the source domain. In this work, to enhance the representative ability, the cluster center of the learned features for each degradation pattern in the source domain is defined as the anchor point for the corresponding degradation in the target domain, as these cluster center features are considered as more representative and generalizable, helping to address the challenge of diverse and unpredictable sample distributions in the target domain.

During the training on the source domain dataset, the degradation features generated by the DAAM for each sample $x_{j}$, denoted as $z_{j}$, is temporarily collected and stored in the forward pass. At the end of each epoch, all collected features are grouped according to their corresponding degradation pattern. For each degradation category $d_{i}$, an epoch-specific anchor $A_{d_{i}}^{E}$ is computed as the arithmetic mean of all features $z_{j}$ whose samples belong to this degradation pattern:

AdiE=1Sdi∑xj∈Sdizj, (30)

where $\left|\;S_{d_{i}}\;\right|$ denotes the number of samples associated with degradation pattern $d_{i}$. This procedure produces a set of anchors $A_{d_{i}}^{E}$ for each epoch E. Upon completing training procedure, the set of anchors associated with the best-performing validation model is retained as final anchors, denoted as $A_{c}$, in subsequent TTA module.

During the model training on the source domain data, the DAM is inactive to block the gradient computation and weight updates. For the training on the target domain data, the DAM is activated to calculate the gradients to update the trainable parameters of the proposed model, which is to achieve the domain adaptation by dynamically correcting the feature distribution.

As shown in Fig. 6B, the DAM projects the input degradation features into higher channels using a 1×1 convolution operation and further captures local spatial information with a 3×3 depthwise separable convolution operation. The nonlinear transformation is able to extract fine-grained features for degraded images in the target domain, as well as reduce the computational complexity. The adaptive features $F_{adapt}$ are obtained by:

$$\begin{array}{c} \begin{array}{c} F_{adapt}=\Psi \left(F_{d}\right),\;\text{where}\;\Psi =\sigma \;\circ \;\mathrm{DWC}\;\circ \;\sigma \;\circ \;{\mathrm{Conv}}_{1\times 1}, \end{array} \end{array}$$ (31)

where $F_{d}$ represents the degradation features, ${\mathrm{Conv}}_{1\times 1}$ represents the convolution operation with a 1×1 kernel, DWC represents the 3×3 depthwise separable convolution, and σ is the Gaussian Error Linear Unit (GELU) activation function.

In addition, the DAM further aggregates informative global features to match the corresponding anchor point by adaptive channel-wise weights from the SENet [46], as in:

$$\begin{array}{c} \begin{array}{c} \mathrm{SE}\left(F_{adapt}\right)=\delta \left({\mathrm{Conv}}_{1\times 1}\circ \sigma \circ {\mathrm{Conv}}_{1\times 1}\left(\text{AvgPool}\left(F_{adapt}\right)\right)\right),\\ F_{\mathrm{se}}=F_{adapt}\times \mathrm{SE}\left(F_{adapt}\right), \end{array} \end{array}$$ (32)

where δ is the sigmoid activation function.

Finally, the final feature of the DAM is obtained using a 1×1 standard convolution operation, as in:

$$\begin{array}{c} F_{dam}={\mathrm{Conv}}_{1\times 1}\left(F_{se}\right). \end{array}$$ (33)

By designing the mentioned DAM architecture, the TTA technique is proposed to train the DAM based on the trained model. Existing work [47] typically fine-tunes the IR model depending only on the target domain data via TTA, with insufficient attention on the learned prior of degradation patterns in the source domain, which leads to catastrophic forgetting and further reduces solution performance [48]. In general, the IR model optimized on the source domain data can generate informative features to support the identification of degradation patterns, which extracts intrinsic and generalizable restoration prior to denoting a shared latent representation of degradation patterns in the target domain. During the TTA procedure, the CORAL loss [49] is improved to guide the feature alignment and correlation between the target and anchor. As described before, during the test procedure with the target domain data, the DAM is activated to perform fine-tuning and inference in a sample-wise manner. In this context, the trainable weights of the DAM are selected to enhance the backpropagation of the gradients, allowing the CORAL loss to tighten the output degradation features between the DAM and the predefined anchor point.

During the testing phase on the target domain datasets, the parameters of the backbone network remain frozen, while only the parameters of the DAM are unfrozen to allow dynamic updates during inference on the target domain. For the input feature $x_{T}$ of the DAM, a forward propagation with the initial weights is first performed, thereby obtaining the corresponding feature representation within the DAM:

$$\begin{array}{c} z_{t}=f_{\theta^{t-1}}^{DAM}\left(x_{T}\right), \end{array}$$ (34)

where $z_{t}$ denotes the feature representation of the DAM, while $f_{\theta^{t-1}}^{DAM}$ represents the computation of the DAM at time step t−1. To avoid the interference caused by the optimization performed on the previous sample, the weights of the DAM are reinitialized for each input sample before the first forward propagation. Subsequently, the distance between *z_t_* and the previously introduced anchor point $A_{c}$ is computed, with CORAL employed for this purpose. The CORAL is described in the following section. The loss $L_{t}$ is formulated as:

$$\begin{array}{c} L_{t}=L_{CORAL}\left(z_{t},\;A_{c}\right). \end{array}$$ (35)

The computed loss is then used to update the parameters of the DAM through gradient descent:

$$\begin{array}{c} \theta_{t}^{DAM}\leftarrow \theta_{t-1}^{DAM}-\alpha_{TTA}\cdot \nabla_{\theta^{\mathrm{DAM}}}L_{t}, \end{array}$$ (36)

where $\theta^{DAM}\in \theta$ denotes the weights of the DAM, while θ represents the weights of the entire model. The above procedure is repeated (5 times in this work) to obtain the final weights $\theta_{T}$, which are then used for the final inference:

I^T=fθTI, (37)

where $f_{\theta_{T}}$ denotes the computation of the entire model with the updated weights $\theta_{T}$ and ${\hat{I}}_{T}$ is the final restored image. The algorithm of the TTA procedure with the DAM is presented in Algorithm S2.

The core idea of the CORAL loss function is to measure the distributional discrepancy of the learned degradation features from samples between the source and target domains by utilizing their covariance matrices. Given the feature matrix $D_{S}$ in the source domain and the feature matrix $D_{T}$ in the target domain, their covariance matrices are computed as follows:

$$\begin{array}{c} C_{S}=\frac{D_{S}^{⊤}D_{S}-\frac{1}{n_{S}}{\left(1^{⊤}D_{S}\right)}^{⊤}\left(1^{⊤}D_{S}\right)}{n_{S}-1}, \end{array}$$ (38)

$$\begin{array}{c} C_{T}=\frac{D_{T}^{⊤}D_{T}-\frac{1}{n_{T}}{\left(1^{⊤}D_{T}\right)}^{⊤}\left(1^{⊤}D_{T}\right)}{n_{T}-1}, \end{array}$$ (39)

where $n_{S}$ and $n_{T}$ denote the number of spatial elements in the given feature maps, respectively, and 1 represents a column vector with all elements equal to one. Finally, the CORAL loss is obtained by normalizing this value:

$$\begin{array}{c} L_{CORAL}=\frac{1}{4dim^{2}}{\left\|C_{s}-C_{t}\right\|}_{F}^{2}, \end{array}$$ (40)

where dim is the dimensionality of the feature representations; ${\left\|∙\right\|}_{F}^{2}$ denotes the squared matrix Frobenius norm.

## Data Availability

The datasets used in this work are publicly available. The code is publicly available at https://github.com/JunyuFan/UDAIR.

## Supplementary Materials

Supplementary 1 Table S1 Figs. S1 to S5 Algorithms S1 and S2
