# Supplementary material for: From Controlled Scenarios to the Real World: Cross-Domain Degradation Pattern Matching for All-in-One Image Restoration
Source: Research (Wash D C). 2026 Mar 27;9:1191. doi: 10.34133/research.1191 (PMC13022318; doi:10.34133/research.1191)
Supplement: Supplementary 1 — Table S1 Figs. S1 to S5 Algorithms S1 and S2 [file research.1191.f1.zip › Supplementary.pdf]

From Controlled Scenarios to the Real-World: Cross-Domain Degradation Pattern Matching for All-in-One Image Restoration

Junyu Fan[1], Chuanlin Liao[2], Endi Xie[1], Dongyue Guo[1], Xiaolin Gou[2], Duan Wei[1], Junyang Hu[1], and Yi Lin[1*]

[1]College of Computer Science, Sichuan University, Chengdu, China.
[2]The National Key Laboratory of Fundamental Science on Synthetic Vision, Sichuan University, Chengdu, China.
[*]Address correspondence to: yilin@scu.edu.cn

Supplementary Information

1 All-in-One Image Restoration Task Overview and Roadmap

Traditional image restoration methods are mostly designed for single degradation scenarios, where each task (e.g., denoising, dehazing, deraining, or low-light enhancement) requires a separate model or specific parameters. Although effective in controlled settings, such Single Degradation Image Restoration (SDIR) methods fail to generalize to unknown or mixed degradations. Multiple Degradation Image Restoration (MDIR) improves generality by handling different degradations within a unified framework, but it still depends on prior knowledge of the degradation pattern and requires task-specific adjustments. All-in-One Image Restoration (AiOIR) has therefore emerged as a more practical solution. It aims to restore images degraded by diverse and unknown patterns using a single framework with unified parameters, without additional priors, as shown in Fig. S1. This paradigm offers clear advantages in real-world applications such as autonomous driving, UAVs, and underwater robotics, where degradations are complex and unpredictable.

However, existing AiOIR studies are still limited to single-degradation datasets, and effective solutions for coupled degradations (e.g., "rain + haze," "haze + low-light") remain underexplored. Due to the nonlinear interaction of degradations, modeling and dataset construction for such cases are highly challenging. Future work should focus on building datasets that include both single and coupled degradations, and on developing models capable of adaptively addressing their complex combinations. This direction is expected to significantly enhance the robustness and practicality of

image restoration model in real-world environments.

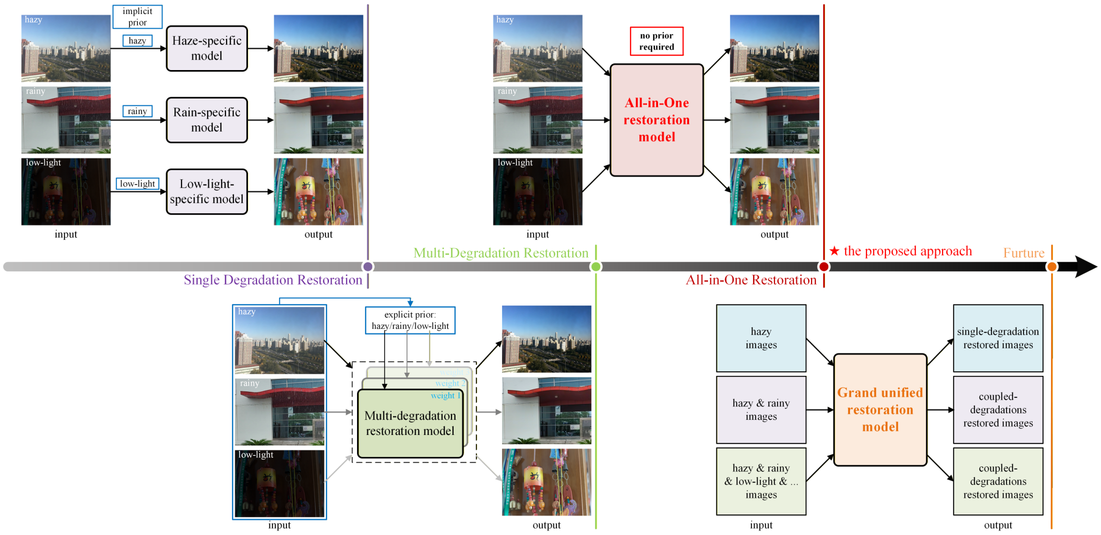

Figure S1: AiOIR task overview and roadmap.

2 Detailed Comparisons

As shown in Fig. S2, the proposed model demonstrates superior capability in detail restoration across diverse degradations compared to competing methods. In the denoising task, only AdaIR, DiffUIR, and the proposed method effectively suppress the noise in severely degraded dark regions, while other baselines either leave noise residues or introduce over-smoothing. In the dehazing task, AirNet, DiffUIR, and the proposed method are the only ones capable of removing haze in the distant regions. Although ROP$^+$ achieves promising performance in removing haze, it inevitably introduces additional artifacts. In the deraining task, ROP$^+$ and CAPTNet still retain noticeable rain streaks, while PromptIR and AdaIR fail to eliminate light streaks. Although AirNet and DiffUIR succeed in removing rain, their results suffer from evident blur artifacts and significant loss of structural information. In the LLIE task, AirNet introduces severe artifacts, whereas CAPTNet suffers from considerable loss of texture details, leading to poor visual quality. In the UIE task, only the proposed method achieves color fidelity close to the reference image, effectively correcting the color cast while preserving fine details. Overall, these comparisons demonstrate that the proposed model excels in identifying degradation patterns and restoring fine details, validating the effectiveness across complex and diverse degradation scenarios.

3 Application Scenarios in Airport Surveillance System

In the airport surveillance system, analysis reveals that visual data are mainly affected by three representative types of degradation, including haze, rain, and partially low-light conditions. These

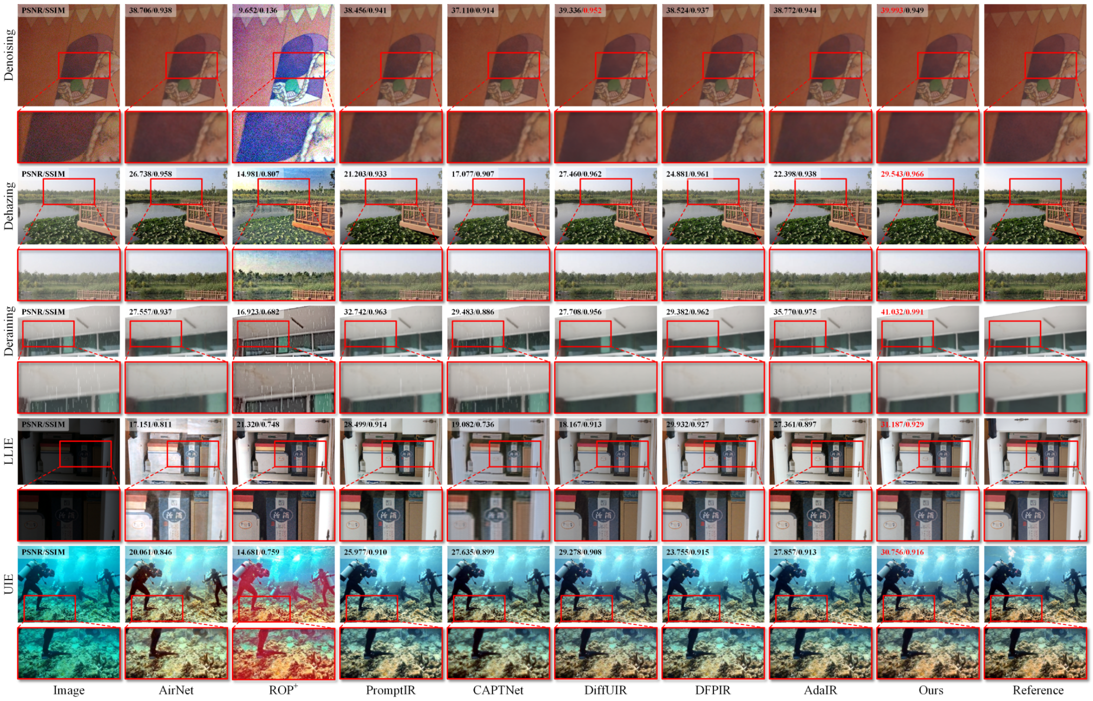

Figure S2: Detailed visual comparisons of different methods.

degradations significantly reduce visibility and obscure structural details, thereby hindering downstream tasks such as object detection, tracking, and situational awareness. As shown in Fig. S3, the proposed model demonstrates superior performance compared with state-of-the-art methods across these diverse conditions. Particularly in low-light conditions, airport surface scenes are often characterized by multiple light sources, backlight, or localized shadows. In such extreme environments, some regions may contain bright light sources or even overexposed areas, which makes it difficult for conventional models to accurately identify the degradation pattern. Benefiting from the CSCL and domain adaptation strategies, the proposed model is able to effectively distinguish these degradation patterns and restore clear images with abundant structural and semantic information. These results highlight that the proposed model provides a unified and robust solution to restore clear images across various degraded conditions in real-world airport environments.

4 Complexity Analysis

To further evaluate the comparative methods, the computational complexities are reported in Fig. S4, concerning the size of trainable parameters and Floating Point Operations Per Second (FLOPs) with respect to the average PSNR (all five tasks on the source domain). In general, the proposed model (UDAIR) harvests a balanced tradeoff between performance and efficiency, i.e., the best PSNR (29.777 dB) with smaller trainable parameters (11.24 M) and moderate FLOPs (205.64 G). The ablation variants also present a similar trend, which confirms the advantages of the proposed

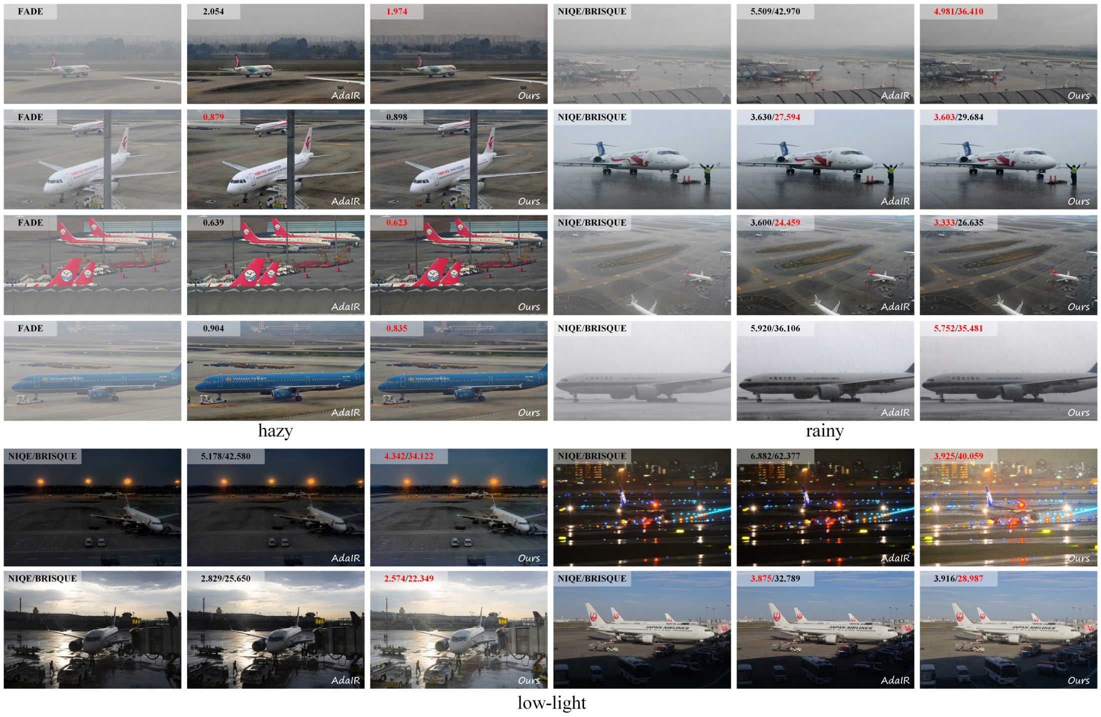

Figure S3: Application results in the airport surveillance scenario.

model architecture and technical modules.

As to the comparative baselines, the AirNet has the smallest model size (only 7.61 M parameters), but suffers from extremely high computational cost with limited inference efficiency (1209.2 G FLOPs). The PromptIR and AdaIR are with 35.59 M / 28.78 M trainable parameters, and 633.62 G / 589.88 G FLOPs, respectively. The DiffUIR also adopts a relatively large architecture with 36.26 M parameters and 398.33 G FLOPs. These models rely on heavy computation to preserve image quality, but such complexity compromises practical deployment. CAPTNet reduces the computational cost to 102.79 G FLOPs with 24.37 M parameters, showing high computational efficiency. However, it struggles to restore fine details and suffers from noticeable quality degradation, especially in the presence of complex degradations.

5 Limitations of Generic TTA on Image Restoration

To investigate the transferability of existing TTA strategies to the image restoration task, a representative method, TENT, is integrated with all selected comparative models. The quantitative evaluation results on the target domain are presented in Table S1.

It is observed that the direct application of TENT fails to yield consistent performance gains for the baseline methods. For the majority of comparative models, performance remains stagnant, and in certain scenarios, distinct degradation is recorded compared to the non-adapted inference. This ineffectiveness implies that TTA strategies originally optimized for high-level vision tasks are

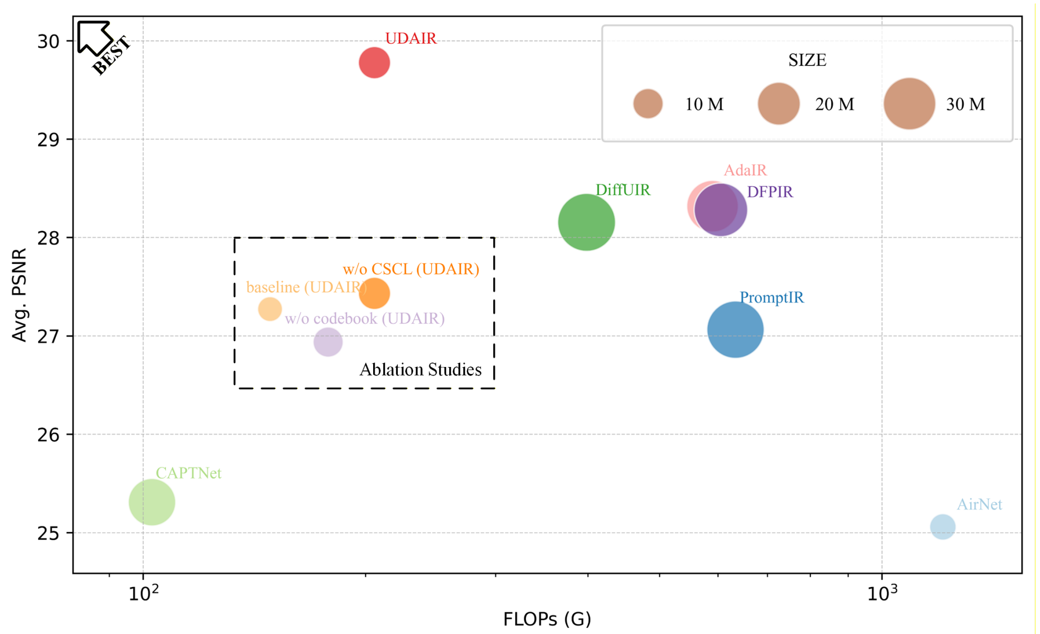

Figure S4: Evaluating efficiency across various metrics on the source domain across all five tasks, models closer to the top-left corner indicate better overall performance.

Table S1: Objective performance comparison of different methods with TTA in comparative analysis, where the best (second-best) performance is indicated in red (blue), ↑ indicates that higher values are better, and ↓ indicates that lower values are better.

Method	Venue	Denoising		Dehazing	Deraining		LLIE		UIE	
		SSIM↑	PSNR↑	FADE↓	SSIM↑	PSNR↑	NIQE↓	BRISQUE↓	UIQM↑	UCIQE↑
AirNet	CVPR'22	0.741	19.570	2.118	0.743	19.287	4.837	17.476	2.752	0.582
PromptIR	NeurIPS'23	0.867	26.064	1.822	0.838	22.142	4.854	15.608	2.737	0.620
CAPTNet	TCSVT'24	0.777	21.390	1.809	0.787	20.011	5.464	22.382	2.804	0.611
DiffUIR	CVPR'24	0.775	20.882	1.705	0.762	20.247	6.049	15.323	2.862	0.619
DFPIR	CVPR'25	0.838	24.986	1.723	0.836	23.240	4.954	16.788	2.801	0.617
AdaIR	ICLR'25	0.865	24.555	1.776	0.835	22.082	4.903	14.387	2.801	0.620
UDAIR	Ours	0.883	27.204	1.585	0.854	23.765	4.814	13.450	2.811	0.626

not directly applicable to the AiOIR task. This phenomenon can be attributed to three critical mismatches:

- TENT operates by minimizing the entropy of model predictions to enhance confidence. While effective for classification tasks where output probabilities are discrete, this objective is unsuitable for image restoration, which fundamentally relies on dense, pixel-wise regression to reconstruct visual details. The entropy minimization does not correlate effectively with the quality of generated images.
- The mechanism of TENT depends heavily on updating the affine parameters of Batch Normalization (BN) layers to align feature distributions. However, many state-of-the-art restoration

architectures favor Layer Normalization (LN) or minimize the use of normalization layers to preserve high-frequency information. Therefore, these models lack the sufficient BN parameters required for TENT to function effectively.

- Existing baselines are designed with fixed weights for static inference and lack specific structural components to accommodate dynamic test-time updates. Forcing adaptation on architectures not designed for it can disrupt pre-trained feature priors, leading to the observed performance decline.

Overall, these experimental results demonstrate that generic TTA methods are suboptimal for the AiOIR task. These limitations motivate the necessity for a specialized adaptation framework. Therefore, in the proposed model, a task-specific TTA strategy and a DAM are developed to specifically address the challenges of feature correction and alignment in image restoration.

6 Efficiency Optimization for Airport Surveillance Systems

To satisfy the operational efficiency requirements for deployment in airport surveillance systems, optimizations and experiments are conducted on the proposed model. In video surveillance scenarios, the variation in image content collected over short durations is limited and highly correlated. Under these conditions:

- Single-sample inference is extended to the parallel input of samples spanning 20-30 consecutive frames (approximately 1 second, depending on the hardware). This extension does not violate the settings and optimization paradigms of TTA; furthermore, it facilitates the introduction of more representative target domain distributions during the optimization process, thereby enhancing the reliability and consistency of TTA updates.

- The number of optimization steps of TTA is reduced to minimize the additional computational overhead incurred by the online optimization process. Since parallel input provides more sufficient and stable target domain statistical information within a single update, the dependence of adaptive updates on the number of steps is significantly decreased, thus maintaining the stability and consistency of restoration performance while reducing the computational load.

- Half-precision computation is introduced during the inference and adaptation phases to further reduce GPU memory consumption and enhance overall inference throughput. This strategy effectively alleviates the resource constraints of online TTA in practical deployment without significantly affecting restoration accuracy, rendering the proposed model more suitable for surveillance systems operating continuously over long periods.

Specifically, 25 images are input in parallel during inference to simulate the data stream per second in video surveillance scenarios. Moreover, the number of steps in the TTA optimization process is reduced from 5 to 3; meanwhile, the calculation precision is adjusted to BFloat16, which preserves the numerical range of FP32 while halving the bit-width.

Experimental results indicate that, on the GeForce RTX 4090 GPU, the average inference time of the proposed model is reduced to approximately 0.04 s/frame after the aforementioned optimizations, with a corresponding increase in processing speed to approximately 25 FPS. While the operational efficiency is significantly improved, a performance decrease of only about 4.4% is observed across five tasks, notwithstanding the lack of surveillance continuity in the evaluation data. If the number of steps is further reduced to 2, the processing speed is increased to approximately 33 FPS while the performance decreases by only approximately 5.3%. Compared to the 24 FPS required for smooth video display, the system handles variations in image content more effectively, further enhancing the stability of restoration results. Furthermore, experiments are conducted using the RTX Pro 6000 GPU based on the state-of-the-art Blackwell architecture. With 2 steps, the processing speed reaches a rate of approximately 60 FPS, which is far higher than the requirements of conventional video streams. This provides sufficient computational margin for real-time deployment and ensures the stability of restoration results at high frame rates.

7 Algorithm of CSCL

In the Algorithm S1, the input degradation feature matrix $F_d \in \mathbb{R}^{\text{batch\_size} \times \text{features}}$ is first reshaped into a task-wise tensor $F_t \in \mathbb{R}^{N_t \times N_s \times \text{features}}$, where N_t and N_s denote the number of tasks and the number of samples per task, respectively. The original features are aggregated within each task by flattening along the sample dimension to obtain $F_g \in \mathbb{R}^{N_t \times (N_s \cdot \text{features})}$. To form positive-negative pairs for contrastive learning, the samples are randomly permuted within each task to produce a shuffled tensor, which is likewise flattened into $F_s \in \mathbb{R}^{N_t \times (N_s \cdot \text{features})}$. Finally, the contrastive loss $\text{loss} = \text{CL}(F_g, F_s)$ is computed to encourage the model to learn the shared intrinsic representations of degradation patterns. The entire procedure executes in a single forward pass and returns the resulting loss for backpropagation.

Algorithm S1: Algorithm of CSCL

Input:

$\mathrm{F_d}$: Degradation features of shape [batch_size, features]

$\mathrm{N_t}$: Number of tasks

$\mathrm{N_s}$: Number of samples per task

CL: Contrastive loss function

Output:

loss: Computed loss value

1: Reshape to task-wise mini-batches:

$F_t = \mathit{reshape}(\mathrm{F_d},\ [\mathrm{N_t},\ \mathrm{N_s},\ \text{features}])$

2: Aggregate original features by task:

$F_g = \mathit{reshape}(F_t,\ [\mathrm{N_t},\ \mathrm{N_s} * \text{features}])$

3: Shuffle samples within each task:

Initialize an empty list: $F_s = [\ ]$

$for\ \ i = 0\ \ to\ \ \mathrm{N_t} - 1:$

$I_p = random_permutation(samples)$

$shuffled = F_t[i,\ I_p,\ :]$

$append\ \ shuffled\ \ to\ \ F_s$

$end\ for$

4: Aggregate shuffled features by task:

$F_s = \mathit{reshape}(cat(*[F_s]),\ [\mathrm{N_t},\ \mathrm{N_s} * \text{features}])$

5: Compute contrastive loss:

$loss = \mathrm{CL}(F_g,\ F_s)$

6. Return *loss*

8 Algorithm of TTA with DAM

In this implementation, as shown in Algorithm S2, the TTA with the DAM is conducted by restricting the adaptation process only to the parameters of the DAM, while freezing all other parts of the network. Given a test image I_t, the model is initialized such that only the parameters θ^{DAM} are set to be trainable. During each of the k adaptation steps, the model produces the predicted image $\hat{I}_i$ together with the latent representation z_i. To align the feature distribution with the anchor point A_c, the CORAL loss is computed as $L_i = \text{CORAL}(z_i, A_c)$, which measures the discrepancy between the second-order statistics of the test features and the anchor. The gradients of L_i are then backpropagated and used to update θ^{DAM}, while all other parameters remain unchanged. After k iterations of adaptation, the final prediction $\hat{I}_t$ is obtained with the updated DAM parameters. This procedure allows the model to adapt to domain-specific shifts in a lightweight and stable manner, while preventing catastrophic updates to the full network during test time.

Algorithm S2: Algorithm of Test-Time Adaptation Procedure with DAM

Input:
$\mathrm{I_t}$: Test image
θ^{DAM}: DAM module parameters
$\mathrm{A_c}$: Anchor point
α: Learning rate
k: Number of TTA step

Output:
$\hat{\mathrm{I}}_\mathrm{t}$: Prediction image with Test-Time Adaptation

1: Initialize model parameters:
for each parameters θ_i in θ:
if θ_i in θ^{DAM}:
set θ_i.requires_grad $\rightarrow$ True
else:
set θ_i.requires_grad $\rightarrow$ False
end for

2: Updated DAM parameters in k steps:
for i in 0 to $k-1$:
a. Feature extraction:
$\hat{I}_i,\ \ z_i = \text{model}(I_t)$

b. Compute CORAL loss:
$L_i = \text{CORAL}(z_i, A_c)$

c. Backpropagate gradients and update DAM parameters:
$\theta_i^{DAM} = \theta_{i-1}^{DAM} - \alpha \nabla_{\theta^{DAM}} L_i$

end for

3. Final prediction with updated DAM parameters:
$\hat{I}_t = \text{model}(I_t)$

9 Limitation of DAAM

Fig. S5 illustrates the two-dimensional feature embeddings produced by DAAM on the synthetic BSD dataset corrupted with additive Gaussian noise at three different levels ($\sigma = 15, 25, 50$). Each noise level forms a well-separated cluster, demonstrating that increasing noise intensity substantially alters the energy distribution and texture statistics of the input images. As σ rises from 15 to 50, the corresponding clusters not only shift but also spread more widely, indicating that higher degradation levels disrupt the consistency of learned representations. Although the clear separation of clusters shows the ability of the proposed DAAM to distinguish different noise levels, it also gently highlights a subtle limitation of DAAM in this work: the underlying energy and texture statistics shift so markedly that current DAAM may not fully maintain invariant representations across all noise intensities. Addressing this could involve designing additional mechanisms for noise-robust feature alignment in future work.

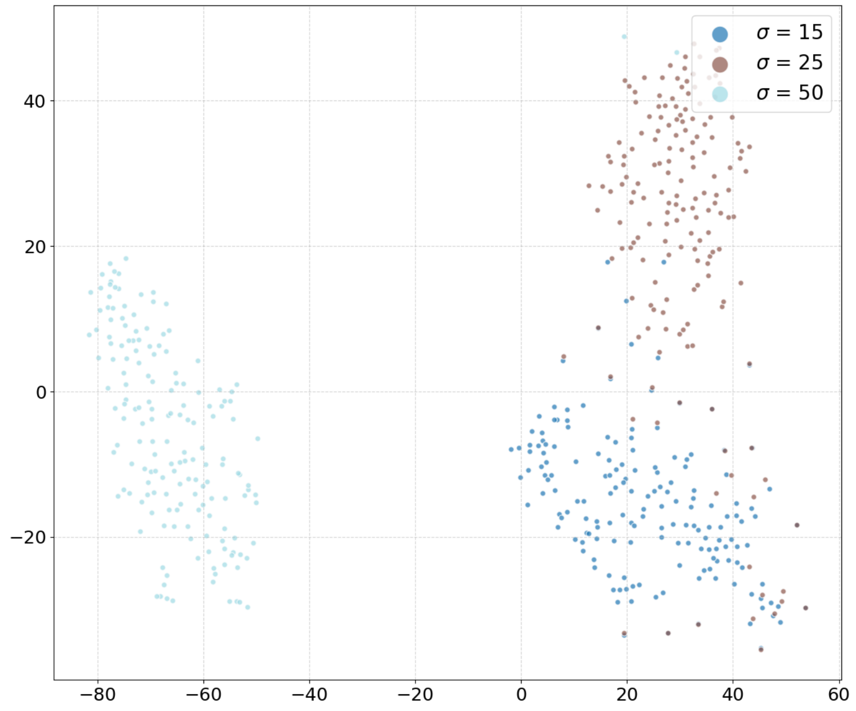

Figure S5: T-SNE visualization of same degradation pattern with different intensities.

10 Dataset Details

The datasets used in this work are publicly available, and the links are provided below:

SIDD: https://abdokamel.github.io/sidd

Polyu: https://github.com/csjunxu/PolyU-Real-World-Noisy-Images-Dataset

OTS/URHI: https://sites.google.com/view/reside-dehaze-datasets

RealRain-1k: https://github.com/hiker-lw/RealRain-1k

LHP-Rain: https://yunguo224.github.io/LHP-Rain.github.io

LOL: https://daooshee.github.io/BMVC2018website

LIME: https://sites.google.com/view/xjguo/lime

UFO-120: https://irvlab.cs.umn.edu/resources/ufo-120-dataset

UIEB: https://li-chongyi.github.io/proj_benchmark.html

For the SIDD and OTS datasets, the sample names of the selected scenes in this work are provided in the GitHub repository.
