# Supplementary figures and images for: From Controlled Scenarios to the Real World: Cross-Domain Degradation Pattern Matching for All-in-One Image Restoration
Source: Research (Wash D C). 2026 Mar 27;9:1191. doi: 10.34133/research.1191 (PMC13022318; doi:10.34133/research.1191)

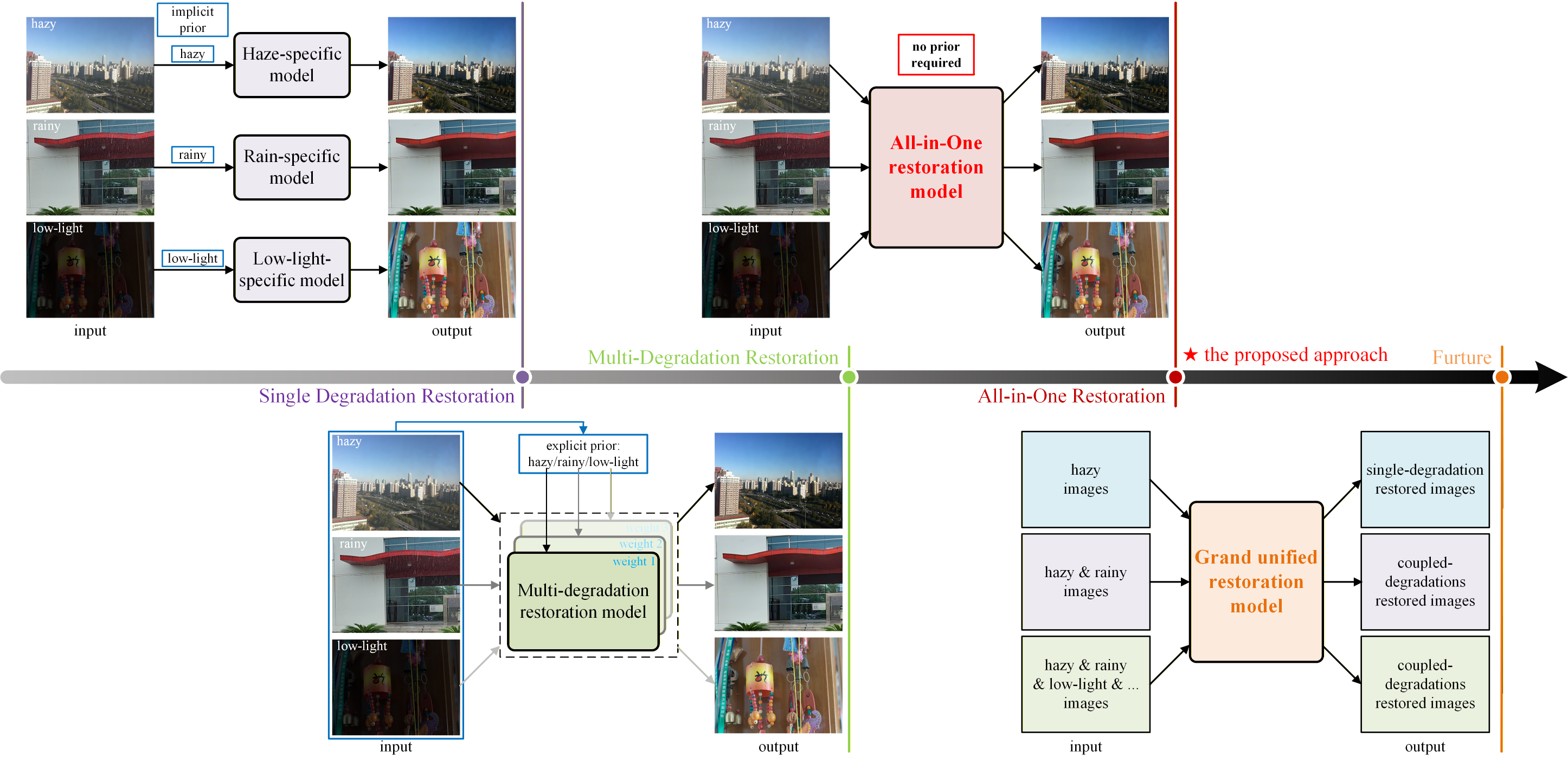

Supplement: Supplementary 1 — Table S1 Figs. S1 to S5 Algorithms S1 and S2 [file research.1191.f1.zip › fig S1.png]

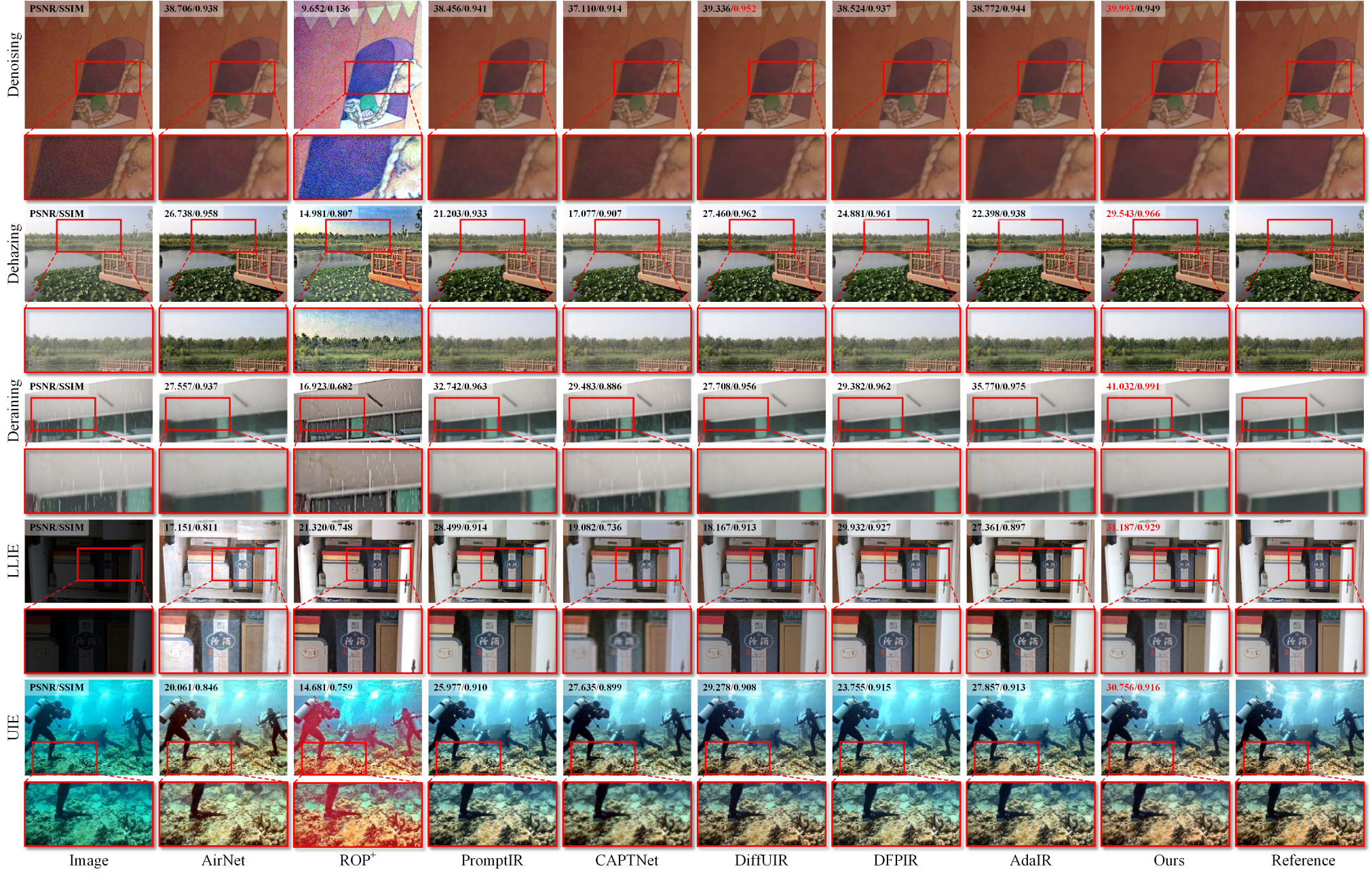

Supplement: Supplementary 1 — Table S1 Figs. S1 to S5 Algorithms S1 and S2 [file research.1191.f1.zip › fig S2.png]

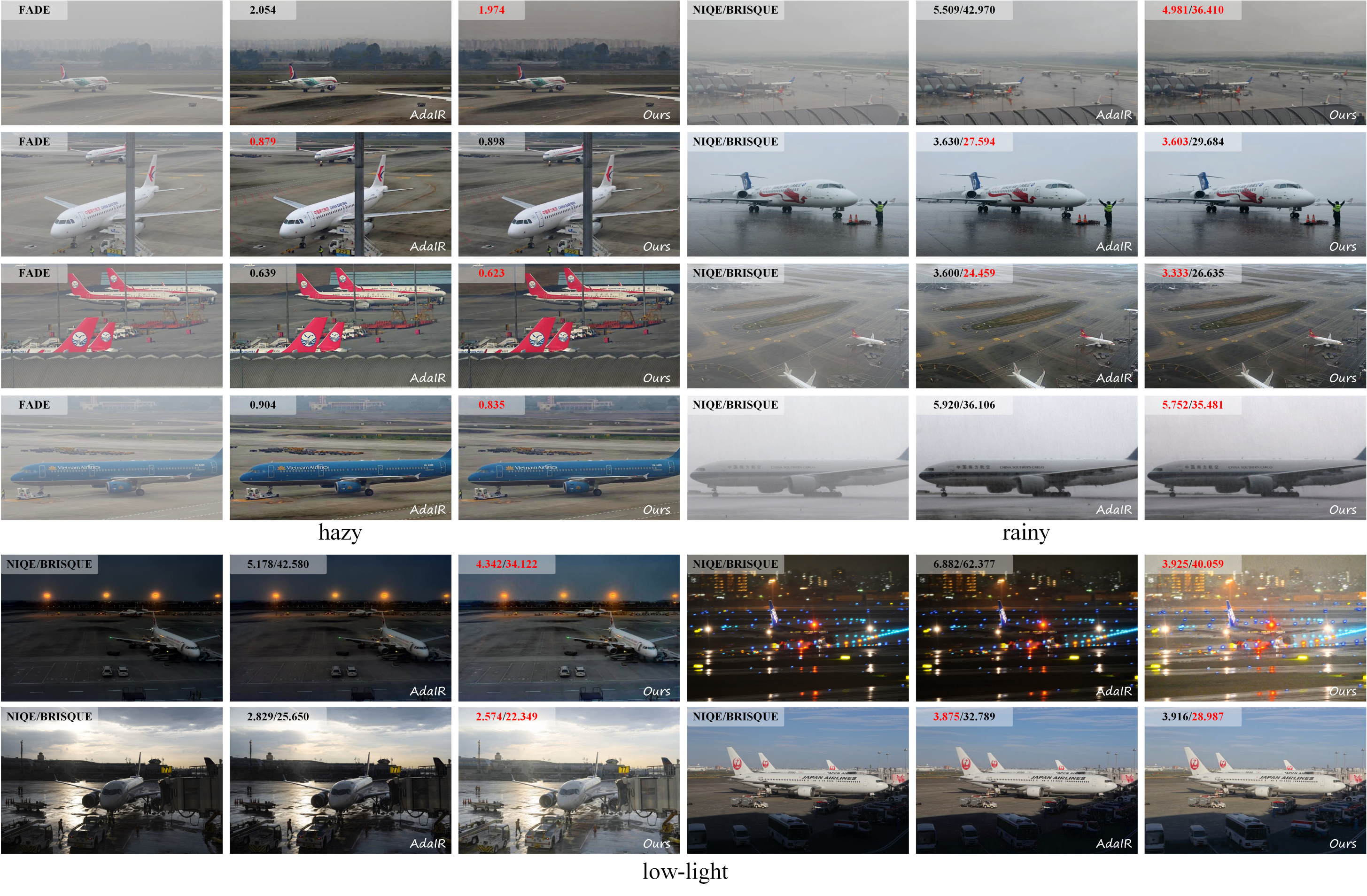

Supplement: Supplementary 1 — Table S1 Figs. S1 to S5 Algorithms S1 and S2 [file research.1191.f1.zip › fig S3.png]

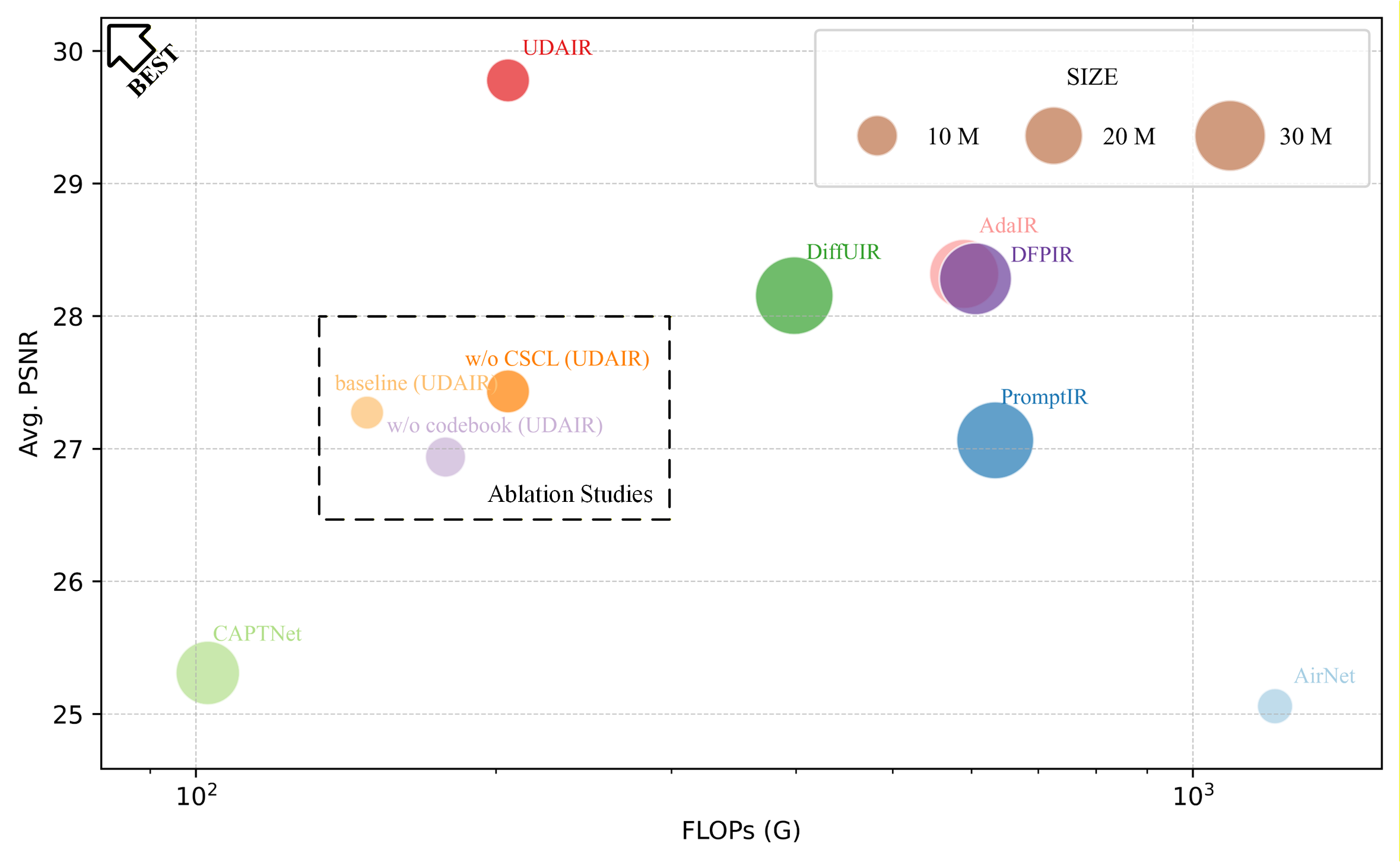

Supplement: Supplementary 1 — Table S1 Figs. S1 to S5 Algorithms S1 and S2 [file research.1191.f1.zip › fig S4.png]

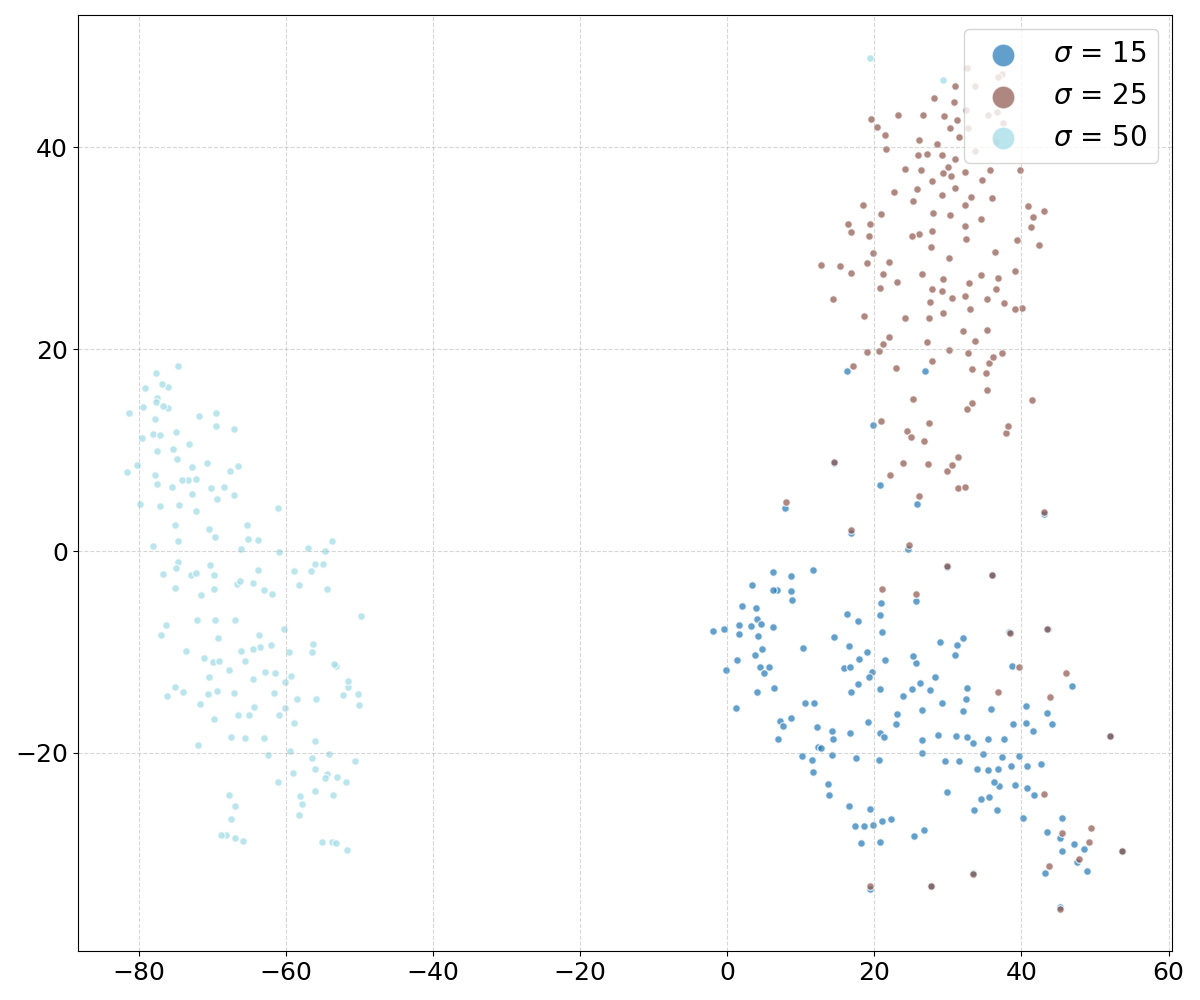

Supplement: Supplementary 1 — Table S1 Figs. S1 to S5 Algorithms S1 and S2 [file research.1191.f1.zip › fig S5.png]
